# Electroacupuncture Ameliorates Knee Osteoarthritis By Rebalancing T Cell Homeostasis as Revealed By Immune Repertoire (IR) Sequencing

**DOI:** 10.2174/0113862073303471240805061026

**Published:** 2024-08-12

**Authors:** Wenrui Jia, Yunan Zhang, Tianqi Wang, Cunzhi Liu, Jianfeng Tu, Guangxia Shi, LingYi Cai, Jingwen Yang, Guangrui Huang

**Affiliations:** 1 School of Acupuncture-Moxibustion and Tuina, Beijing University of Chinese Medicine, Beijing 102488, China;; 2 School of Life Science, Beijing University of Chinese Medicine, Beijing 102488, China

**Keywords:** Electro-acupuncture, knee osteoarthritis, immune repertoire, t cell receptor, neutrophil, transcriptome

## Abstract

**Background:**

In this study, we used immune repertoire (IR) sequencing technology to profile the diversity of peripheral blood T cell receptors and used transcriptomics to profile the gene expression of peripheral blood neutrophil mRNA in patients with mild-moderate knee osteoarthritis (KOA) before and after electroacupuncture (EA) treatment.

**Methods:**

An 8-week intervention with EA was performed on 3 subjects with KOA. IR sequencing of complementarity determining region 3 (CDR3) was performed using RNA extracted from peripheral blood T cells of KOA subjects prior to and at the end of the intervention, as well as healthy volunteers (controls) who matched the subjects in sex and age. Neutrophils were extracted from the plasma of healthy individuals, pretreatment patients, and posttreatment patients for further transcriptome sequencing.

**Results:**

The D50, diversity index (DI), and Shannon entropy values of circulatory T-cells were significantly lower in pretreatment KOA patients compared to healthy controls. Posttreatment KOA samples displayed significant decreases in serum proinflammatory factors, IL-8 and IL-18 (*P* < 0.01), as well as a substantial reduction in serum matrix MMP-3 and MMP-13 (*P* < 0.01, *P* < 0.05). Transcriptome analysis revealed that the expression of CXCL2, IRF8, and PEAR1 (*P* < 0.05) was significantly higher in patients before the treatment than in the healthy population and was significantly down-regulated after the treatment. In contrast, the expression of SMPD3 (*P* < 0.05) showed the opposite trend.

**Conclusion:**

EA may alleviate KOA by rebalancing T-cell homeostasis and improving systemic inflammation. At the same time, EA treatment can significantly enhance TCR diversity, reduce levels of proinflammatory factors, and increase levels of anti-inflammatory factors, thereby achieving therapeutic effects.

**Clinical Trial registration:**

NCT 03366363.

## 1 INTRODUCTION

Knee osteoarthritis (KOA) is one of the most common musculoskeletal diseases, and middle-aged and older adults are the most vulnerable, especially women [1]. A systematic review showed a disease prevalence of approximately 27.3% in women and approximately 21.0% in men [2]. A preponderance of research has been performed to examine the pathogenesis of KOA, but its etiology remains unclear. Based on previous studies, KOA is considered to comprise a degenerative change of articular cartilage and its peripheral tissues induced by multiple stimuli. Increasing evidence in recent years suggests that immune dysfunction and inflammation play important roles in the pathogenesis of KOA, which is related to both innate and adaptive immune disorders characterized by inflammation and immune reactions, as indicated by previous research [3-5]. Factors promoting the pathogenesis of KOA have been identified in previous studies, including the production of damaging cytokines by circulatory T-cells, unhealthy aging of T-cells, and the entry of healthy aged T-cells driven by any other factor into an imbalanced status [6]. KOA patients have a prolonged status of chronic systemic inflammation with elevated levels of circulatory inflammatory factors [7]. Research has indicated that KOA pathogenesis is more significant than imaging alterations brought on by mild inflammation, and that inflammation is systemic rather than localized in the knee joint [8, 9]. Therefore, KOA pathogenesis, seems to be closely correlated with systemic immune dysfunction and inflammatory status.

Both localized inflammation and systemic inflammation are manifested in this process. Localized inflammation of the knee joint is mainly manifested by joint swelling, pain, stiffness, and limitation of movement. This is due to the degeneration of articular cartilage and the formation of osteophytes. In addition, inflammation of the synovium can occur, leading to an increase in joint fluid, which can cause swelling of the joint. A systemic inflammatory response is also important. This is mainly reflected in the increased expression of some systemic inflammatory factors, such as interleukin-1β (IL-1β) and tumor necrosis factor-α (TNF-α) [10]. These factors induce the expression of more inflammatory factors, enhance the NF-κB signaling pathway, and ultimately lead to destruction, senescence, and inflammatory changes in the knee cartilage. Neutrophils play a crucial role in the inflammatory response. They are the main phagocytes in the blood, with strong deformation wandering ability and phagocytic activity [11]. In an acute inflammatory response, neutrophils are the first leukocytes to reach the site of infection or injury [12, 13]. The recruited neutrophils will attempt to destroy the inflammation-causing factors in order to resolve and heal the inflammation eventually. In order to investigate the mechanism of EA in treating KOA from the perspective of immunity and inflammation, we chose T cells (the key immune cells of KOA) and neutrophils (the key inflammatory cells of KOA) in peripheral blood as the main object of observation.

The present treatment against KOA is centered on the relief of pain, aiming to maintain or facilitate joint function and improve the quality of life for patients. The current clinically adopted principle is symptomatic treatment for mild-moderate disease and surgery for severe disease [14, 15]. Acupuncture is considered a potential nondrug intervention preventing or delaying the pathogenesis and progression of KOA [16]. Research has confirmed that 8 weeks of treatment with manual acupuncture or EA can ameliorate knee joint function in patients with mild to moderate KOA and relieve joint pain. This effect is mediated partially by changes in the inflammatory factors, such as TNF-α, IL-1β, and IL-13 [17]. Other studies have revealed that EA is able to regulate systemic immunity and cure some immune disorders, thereby treating some of the diseases correlated with immunity or inflammation [18].

The immune repertoire (IR) is defined as the sum of receptor diversities in antigens of all B-cells and T-cells in the circulation of a certain individual at a certain designated time point [19]. The use of IR sequencing can generally evaluate the diversity of the immune system, monitor dynamic changes in IR diversity, systematically analyze the mechanism of immune regulation, and profoundly explore the relationship between IR and diseases [10, 20].

Eight weeks of treatment with EA, as shown by our preliminary study, effectively ameliorated symptoms, such as knee pain, stiffness, and functional limitations in patients with KOA, compared to manual acupuncture or sham acupuncture [21]. Therefore, this study was based on the results of our preliminary study to further explore the mechanism of EA in treating KOA, aiming to answer the issue of whether EA treatment promotes the body to achieve immune balance by correcting systemic immune disorders and recovering the diversity of T-cell receptors (TCRs), relieving inflammation and achieving its therapeutic effect. Therefore, we used IR sequencing, transcriptome sequencing, and ELISA detection to observe changes in the diversity of TCRs, the expression of neutrophil mRNA and serum inflammatory factors in peripheral blood in both healthy female volunteers (controls) and female KOA patients before and after EA intervention to provide a scientific basis for the mechanism of EA in treating KOA.

## 2 MATERIALS AND METHODS

### 2.1 Subject Enrollment and Sample Collection

**Fig. (1):**
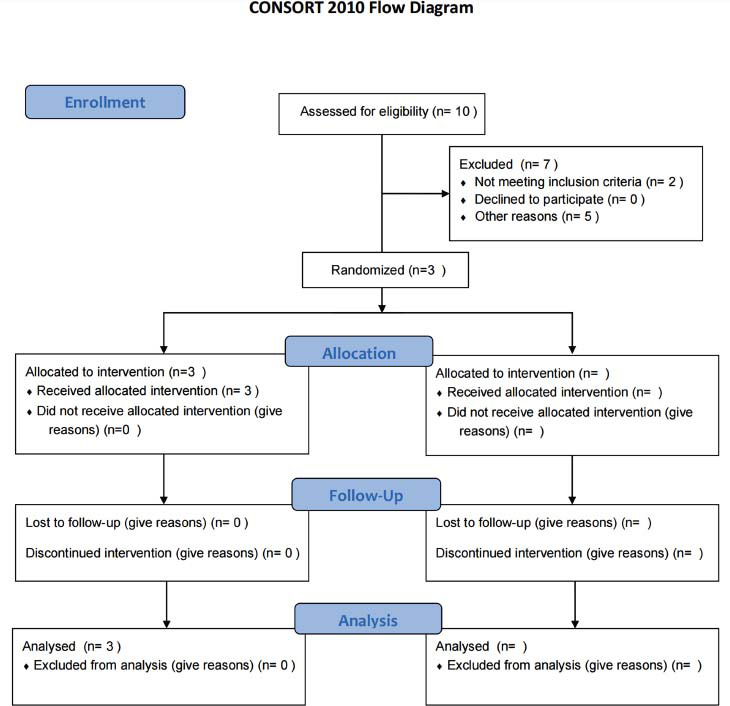
CONSORT flow diagram.

Three patients diagnosed with knee osteoarthritis (KOA) based on the American College of Rheumatology criteria were recruited using various recruitment methods (Fig. **1**) [22, 23]. The inclusion criteria for KOA subjects were as follows: ① female, aged 57-67 years; ② BMI 18.5~26.9; ③ experiencing chronic knee pain (unilateral or bilateral) for the past 6 months; ④ radiographic confirmation of KOA (Kellgren-Lawrence grade II or III); ⑤ average pain intensity of 4 or higher on a numerical rating scale (NRS) in the last week; and ⑥ providing written informed consent. The exclusion criteria were: ① history of knee arthroplasty or awaiting knee surgery for either knee; ② knee arthroscopy in the last 12 months or intra-articular injection within the previous 4 months; ③ knee pain caused by other conditions such as autoimmune diseases, infection, malignant tumors, trauma, fracture, joint bodies, severe joint effusion, lumbosacral vertebrae disease, *etc*.; ④ presence of serious acute or chronic organic diseases or psychiatric disorders; ⑤ blood coagulation disorders; ⑥ pregnancy or breastfeeding; ⑦ received acupuncture treatment in the last 3 months; ⑧ participated in other clinical trials in the past 3 months; ⑨ cardiac pacemakers, metal allergies or needle phobia and ⑩ take other medications, such as antihypertensive drugs, hypoglycemic drugs, *etc*.

To assess pain, stiffness, and function in patients with KOA, the researchers used the Western Ontario and McMaster Universities Osteoarthritis Index (WOMAC) and the numerical rating scale (NRS) [24]. KOA participants completed the questionnaires at the beginning of the study and at the end of the 8th week.

The inclusion criteria for healthy subjects were: ① overall normal results in routine physical examinations (including blood, urine, and stool tests, liver and kidney function, and blood lipid tests); ② female, aged 57-67 years; ③ BMI 18.5~26.9; ④ no unilateral or bilateral chronic knee pain and the Kellgren-Lawrence grade 0; and ⑤ written informed consent. The exclusion criteria were: ① infected with hepatitis B virus, hepatitis C virus, HIV, or Treponema pallidum; ② documented chronic diseases or abnormal medical indicator in medical reports; ③ pregnant or breastfeeding; ④ a history of alcohol or drug abuse; ⑤ take other medications, such as antihypertensive drugs, hypoglycemic drugs, *etc*.; and ⑥ participating in other clinical studies.

This trial was approved by the research ethical committees of Beijing Hospital of Traditional Chinese Medicine Affiliated to Capital Medical University (2019BL02-011). After explaining the study to the participants, they all signed written informed consent forms. The study was registered at the China Clinical Trial Registry with the registration number NCT 03366363 on November 20, 2017, before recruiting the first participant.

### 2.2 EA Intervention

The EA (Electroacupuncture) treatment follows a semi-standardized approach [10]. The acupuncture prescription includes five obligatory acupoints and three adjunct acupoints. The obligatory acupoints are dubi (ST35), neixiyan (EX-LE5), ququan (LR8), xiyangguan (GB33), and an ashi point (the point where the patient feels the most pain). The acupuncturists choose the adjunct acupoints based on traditional Chinese medicine principles. If the patient experiences pain in the anterior aspect of the affected knee joint, it indicates yangming meridian syndrome. In this case, three adjunct acupoints are selected from futu (ST32), liangqiu (ST34), heding (EX-LE2), zusanli (ST36), and fenglong (ST40). If the pain occurs in the medial aspect of the affected knee joint, it indicates three-yin meridian syndrome. In this case, three adjunct acupoints are selected from xuehai (SP10), yingu (KI10), yinlingquan (SP9), xiguan (LR7), sanyinjiao (SP6), taixi (KI3), taichong (LR3), and gongsun (SP4). If the pain occurs in the posterior aspect of the affected knee joint, it indicates Taiyang meridian syndrome. Three adjunct acupoints are then chosen from Weiyang (BL39), Weizhong (BL40), Chengshan (BL57), and Kunlun (BL60). If the pain occurs on the lateral aspect of the affected knee joint, it indicates shaoyang meridian syndrome. Three adjunct acupoints are then chosen from fengshi (GB31), yanglingquan (GB34), waiqiu (GB36), xuanzhong (GB39), and zulinqi (GB41). If more than two aspects are affected, three adjunct acupoints are chosen from those corresponding to the relevant syndromes. The acupoints are determined based on the WHO Standard Acupuncture Locations. For patients with bilateral osteoarthritis, both knees are treated with acupuncture. For patients with unilateral osteoarthritis, only the affected knee is needled. The needles are manually stimulated for at least 10 seconds to achieve de qi, which is a combination of sensations like soreness, numbness, distention, and heaviness. Then, electrodes from an EA apparatus are attached to the needle handles at LR8 and GB33, as well as to two other adjunct acupoints by the research assistant. The EA apparatus used is the HANS-200A acupoint nerve stimulator from Nanjing Jisheng Medical Co, Ltd. The wave is set at 2/100 Hz, and the electric current is gradually increased until the needles start to vibrate slightly. The EA therapy consists of 24 sessions, each lasting 30 minutes, administered over 8 weeks (typically three sessions per week).

### 2.3 Collection of Venous Blood Samples and Separation of Lymphocytes and Neutrophil

Venous blood was collected from healthy volunteers (controls) and KOA subjects before and after EA intervention. Fifteen milliliters of venous blood were collected and kept in EDTA anticoagulant tubes on ice. Cells were separated within 2 hours using gradient density centrifugation with PolymorphprepTM reagent. The PolymorphprepTM reagent was removed from the 4°C refrigerator in darkness, allowed to return to ambient temperature, and added to 7 mL of each of the three 15 ml centrifuge tubes. All blood samples were dripped carefully along the wall of the centrifuge tube onto the surface of the PolymorphprepTM reagent. Gentle centrifugation (500 g for 35 min) was performed, followed by observation for two-layer stratification of cells, and a second centrifugation was required in the absence of stratification. Mononuclear cells on the upper layer and neutrophils on the lower layer were aspirated respectively, resuspended in an equal volume of 0.5 × PBS solution, and centrifuged at 500 g for 5 mins, and the supernatant was discarded. Cell precipitate was added to red blood cell lysate, mixed well and cooled on ice for 15 min, during which it was shaken 2-3 times. Then, samples were centrifuged again at 500 g for 5 minutes, and the supernatant was discarded. One milliliter of TRIzol reagent was added, blown and shaken to fully dissolve the precipitate. The solution was stored in a -80°C freezer until RNA extraction.

### 2.4 Extraction of RNA

Samples were removed from the -80°C freezer, thawed, and incubated at room temperature for 5 min, added to a mixture of chloroform -TRIzol (v/v: 200μl/1 ml), strongly vortexed for 15-20 seconds, incubated for 2-3 minutes, and centrifuged at 4°C for 15 minutes. The upper aqueous phase was transferred into a 1.5 ml RNase-free centrifuge tube, added to a solution of isopropanol-TRIzol (v/v: 0.5 ml/1 ml), mixed well by inversion, allowed to sit at ambient temperature for 10 minutes, centrifuged at 12000 g at 4°C for 10 min with the supernatant discarded, and added to an equal volume of 75% ethanol. The bottom of the tube was gently flicked to suspend the precipitate. The suspension was centrifuged at 7,500 g at 4°C for 5 minutes, and the supernatant was discarded. The precipitate was air-dried for 3 minutes and completely dissolved in 50 μl of DEPC water, followed by quantification with a NanoDrop.

### 2.5 Determination of RNA Concentration and Quality Inspection

An aliquot of 5 μl RNA solution was used to determine the concentration of RNA extracted with a nucleic acid analyzer. Five microliters of sample were mixed with 1 μl of nucleic acid electrophoresis stain and underwent electrophoresis using a 2% agarose gel. Pictures were taken with a nucleic acid imager. Again, samples that qualified based on primary inspection underwent microporous electrophoresis on RNA to detect its completeness using an Agilent 2100.

### 2.6 Establishment of IR

The automated library building kit (iRepertoire, Inc. Huntsville, AL, USA) was removed from the 4°C refrigerator, followed by mixing well with 10 inversions. A total of 500 ng of RNA was mixed well with 1.6 μl of Qiagen reverse transcriptase, replenished with DEPC water to a volume of 20 μl, and transferred to the kit. The kit was placed into the gene amplification instrument to automatically establish IR. At the end of the reaction, 37 μl of nucleic acid-free water was used to repeatedly blow the magnetic beads 30 times to completely resuspend the magnetic beads when the liquid appeared rubiginous. The suspension was allowed to stand for 2 minutes at room temperature, and the kit was placed on an iRep magnetic frame. The supernatant was slowly removed while avoiding removing the magnetic beads. IR was preliminarily quantified using a NanoDrop. After the IR concentration was homogenized, sequencing was performed using the Illumina MiSeq PE250 platform.

### 2.7 Analysis of Raw Data

**Fig. (2):**
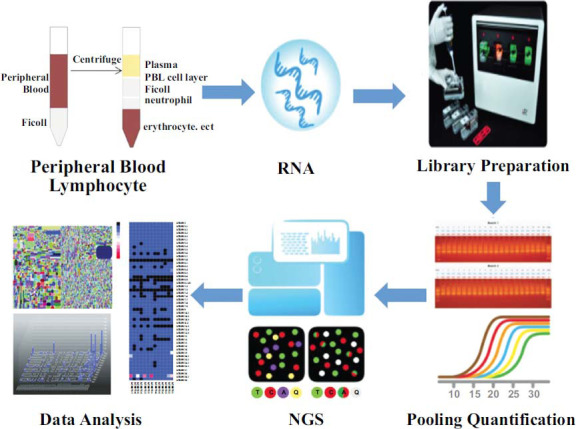
**The workflow of peripheral blood-based TCR repertoire discovery.** RNA extracted from peripheral blood T cells was used for amplification. PCR and library preparation were fully automated in a closed system. After amplification, libraries were pooled, quantified, and sequenced using next-generation sequencing (NGS). Data were analyzed using iRweb’s bioinformatics platform.

Using the IMGT/GENE-DB database, we compared the sequences using the V-, D-, and J genes of the TCR β germline. The analysis was conducted using the Smith-Waterman algorithm of the iR-map line, with visualization in iRweb (iRebertoire, Inc. AL, USA). The data analysis included information on peptide sequence, uCDR3 (unique CDR3), shared CDR3, and the usage of V and J genes (Fig. **2**).

### 2.8 Transcriptome Sequencing

The quality of total RNA was selected as the starting sample for mRNA sequencing. The quality of total RNA was determined by Agilent 2100 BioAnalyzer. The ratio of 28S to 18S RNA was greater than or equal to 1.5:1. The quality of the samples should be RIN ≥ 7 by Agilent 2100 BioAnalyzer, the ratio of 28S to 18S RNA should be greater than or equal to 1.5:1, and the starting amount should be in the range of 0.1ug. The starting Total RNA was accurately quantified using the QUBIT RNA ASSAY KIT. The product was purified with 144uL of AMPure XP Beads and resuspended with 60µL of Nuclease free water, and 55.5µL of the product was removed for the next step; the product was run through Program5 and Program6 on the PCR instrument, and then 100uL of AMPure XP Beads were purified and resuspended with 52.5µL of Resuspension Buffer. Resuspension Buffer: resuspend with 22µL of AMPure XP Beads, and remove 23µL for the next step; run Program7 on the PCR instrument, and purify with 45µL of AMPure XP Beads, followed by 23µL of AMPure XP Beads. Run Program7 on the PCR instrument, then 45µL was purified with AMPure XP Beads, and finally resuspended with 23µL of Resuspension Buffer, and 20µL was taken out for the next step; Prepare freshly prepared NaOH and adjust its concentration to 0.1N; take 0.1N NaOH (10µL) and 2nM library (10µL) and mix vortex.s for mixing vortex, centrifuge and leave at room temperature for 5min, then place on ice; Then add 20µL of denatured single-stranded DNA Library to 980µL of pre-cooled HT1 (Hybridisation buffer) to achieve a final library concentration of 20pM, and place on ice; Cluster the Flowcell and prepared library on a cBot, *i.e.*, molecules in the library are clustered with primers immobilized on the Flowcell. The molecules in the library are combined with the primers fixed on the Flowcell for bridge PCR amplification and then sequenced on the corresponding sequencing platform. The Flowcells are transferred to the sequencing platform and prepared for sequencing. According to the different types of sequencing and the length of sequencing, the correct Recipe is selected for sequencing, and the sequencing cycle is directly related to the length of sequencing. Before the sequencing procedure is run formally, first judge whether the signals of A, T, C, and G bases on each lane are normal according to the First Base Report so as to judge whether the sequencing primers are normal, and then judge whether the sequencing primers are normal.

### 2.9 Detection of Inflammatory Factors

Three milliliters of blood were collected into a procoagulant tube at baseline and after 8 weeks of treatment. The tubes were then centrifuged at 2,500 rpm for 5 minutes. The resulting serum was stored at -80 °C for future use. For the cytokine assay, a Bio-Plex Pro multiassay technology by Bio-Rad Laboratories (Hercules, CA) was utilized. This system utilizes fluorescently dyed beads that are conjugated with monoclonal antibodies to detect specific cytokines. In this case, eleven target cytokines (IL-8, IL-18, IL-1β, TNF-α, MCP-1, IL-10, IL-13, and IL-4) were measured. The beads were incubated with the collected samples, followed by the addition of a secondary biotinylated antibody. The sample was then analyzed using a dedicated flow cytometer equipped with two lasers and associated optics. This allowed for the measurement of the different molecules bound to the surface of the beads and the subsequent determination of cytokine concentrations. To measure the expression levels of MMP-3 and MMP-13, human enzyme-linked immunosorbent assay (ELISA) kits from RayBiotech (Atlanta, GA, USA) were utilized. Additionally, the CCL5 ELISA kit from R&D Systems (Minneapolis, MN, USA) was used to analyze CCL5 levels. All experimental procedures followed the instruction manual. For optical density measurements, an automatic ELISA reader (Sunrise; Tecan, Mannedorf, Switzerland) was employed, and readings were taken at a wavelength of 450 nm.

### 2.10 Statistical Analysis

Statistical analysis was performed using SPSS software (SPSS v25.0, SPSS Inc., Chicago, Illinois, USA). The normality of the distribution of the variables was tested using the Shapiro–Wilk test. The paired sample t-test procedure and one-way ANOVA were used to analyze variables with a normal distribution. Abnormally distributed variables were analyzed using the Kruskal-Wallis test. Enumeration data were tested by chi-square test. Measurement data are expressed as the mean and standard deviation (mean ± SD), and enumeration data are expressed as a percentage. *P* < 0.05 was considered a statistically significant difference.

## 3 RESULTS

### 3.1 Subject Characteristics and Clinical Outcomes

All participants in the study were women. No notable variations in demographic factors such as age, sex, and BMI were observed between the participants with knee osteoarthritis (KOA) and the healthy individuals (Supplementary Table **1**). Among KOA patients, there were no significant differences in pain, stiffness, function, or total WOMAC and NRS scores between the pretreatment (week 0) and posttreatment (week 8) assessments. Nonetheless, a decreasing trend was observed in the average scores of WOMAC pain, stiffness, function, and total scores as well as NRS scores (Supplementary Table **2**).

### 3.2 TCR Repertoire

#### 3.2.1 High-Throughput Sequencing of the TCR Repertoires

**Fig. (3):**
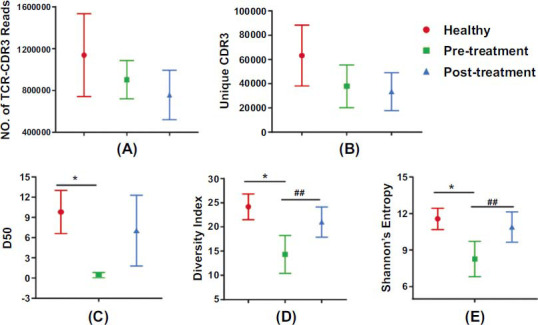
The diversity of TCR CDR3 repertoire. (**A**) The total number of TCR CDR3 reads. (**B**) Unique CDR3 index between three groups. (**C**) D50 index between three groups. (**D**) Diversity index between three groups. (**E**) Shannon's Entropy between three groups. * *P* < 0.05, ** *P* < 0.01.

Using high-throughput sequencing, we analyzed the T cell receptor (TCR) repertoires in T cells obtained from the blood of three groups: healthy individuals (healthy group), patients with knee osteoarthritis (KOA) before electroacupuncture (EA) treatment (pretreatment group), and KOA patients after EA treatment (posttreatment group) (Fig. **3A**, **B**). We focused on the complementary determining region 3 (CDR3), which is the most diverse segment of the TCR beta chain and results from the recombination of variable (V) and constant (C) genes. To capture the CDR3 regions, we performed a nested PCR amplification using gene-specific primers, targeting the variable and constant regions on peripheral blood samples. The resulting PCR products were sequenced using the Illumina MiSeq PE250 platform. On average, 932333.67 reads were obtained from each sample by sequencing the TCR beta chain (Supplementary Table **3**).

#### 3.2.2 Analysis of TCR Diversity

Three indexes, including diversity index (DI), Shannon entropy, and D50, were used to reflect the overall diversity results of the sample. The ranges were 0~13.82 for Shannon entropy (based on the assumption that the maximum number of clone types was one million) and 0~50 for either DI or D50. The value of D50 represents the percentage of the first 50% of a single T-cell clone in total CDR3. D50 is a measurement of the uniformity of T cell clones, and a low D50 value means that IR is biased by large clonal expansion. Higher values in these three indexes suggest high clonal diversity of the sample. It was suggested by D50, DI, and Shannon Entropy that the CDR3 diversity of receptors of peripheral T-cells decreased significantly compared to healthy controls (*P <* 0.05). Compared to values prior to treatment in KOA patients, posttreatment values showed a highly significant rise in both the diversity index of TCRs and Shannon entropy (*P <* 0.01) (Fig. **3C**-**E**).

#### 3.2.3 Distribution Characteristics of CDR3 Length

**Fig. (4):**
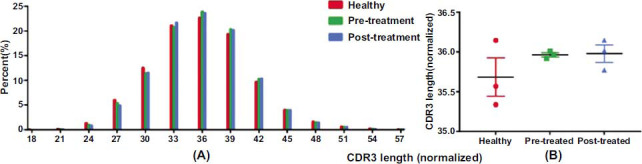
Length distribution of CDR3. (**A**) The distribution of CDR3 lengths in the Healthy, Pre-treatment, and Post-treatment groups. (**B**) The average CDR3 length in the Healthy, Pre-treatment, and Post-treatment groups.

The length distributions appeared to be consistent among the three groups (Fig. **4**).

#### 3.2.4 Differences in Usage and Trim Frequency of Gene V in Each Group

**Fig. (5):**
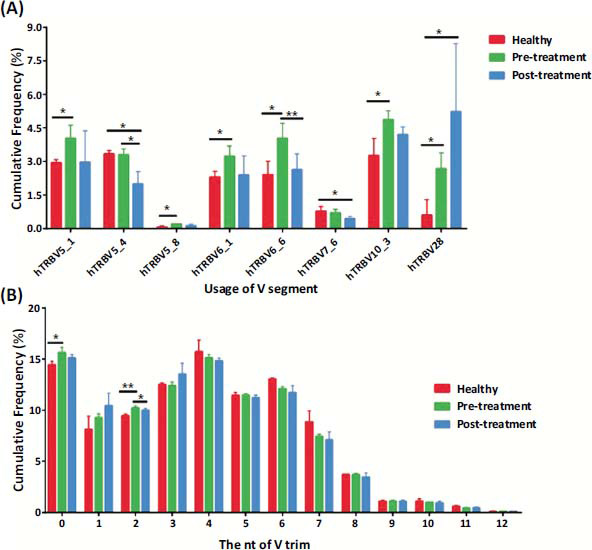
V gene usage and trim in samples of three groups by outcome. (**A**) The frequency of V gene usage in subjects from the healthy control group (red), pretreatment group (green), and posttreatment group (blue)showed only significant differences. (**B**) Frequency of V gene trimming in subjects from the healthy control group (red), pretreatment group (green), and posttreatment group (blue). *P < 0.05, **P < 0.01.

The results showed a marked increase in the usage frequency of gene V, including V5_1, V5_8, V6_1, V6_6, V10_3, and V28, in KOA patients compared to healthy controls, with a significant difference (*P <* 0.05). An obvious decrease in the usage frequency of V6_6 was observed in KOA patients post-EA compared to pretreatment data, with an extremely significant difference (*P <* 0.01) (Fig. **5A**). The results in KOA patients showed an obvious increase in the frequency of two trims in gene V compared to the healthy controls (*P <* 0.01). Compared to pretreatment data in KOA patients, the frequency of two trims in gene V decreased significantly post-EA (*P <* 0.05) (Fig. **5B**).

#### 3.2.5 Difference in Trim Frequency of Gene J in Each Group

**Fig. (6):**
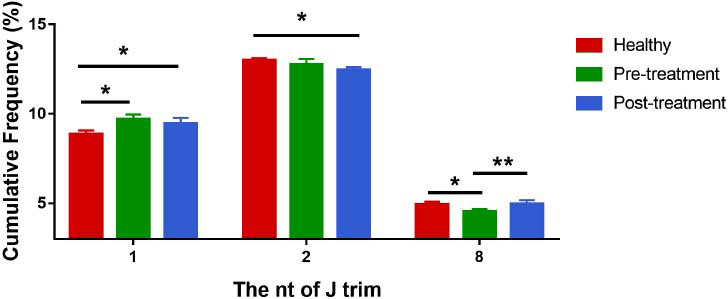
J gene trim in samples of three groups. Frequecy of J gene trimming in subjects from the healthy control group (red), pretreatment group (green), and posttreatment group (blue), showing only significant differences. *P < 0.05, **P < 0.01.

Results showed an obvious increase in the frequency of one trim of gene J in KOA patients compared to healthy controls (*P <* 0.05). A decreasing tendency was observed in the frequency of one trim of gene J in KOA patients post-EA compared to their pretreatment data, despite the absence of a significant difference. Compared to the healthy controls, KOA patients exhibited an obvious decrease in the frequency of eight-time trims of gene J (*P <* 0.05), which increased post-EA with an extremely significant difference in contrast to pretreatment data (*P <* 0.01) (Fig. **6**).

#### 3.2.6 Difference in Nucleotide Addition Frequency in Each Group

**Fig. (7):**
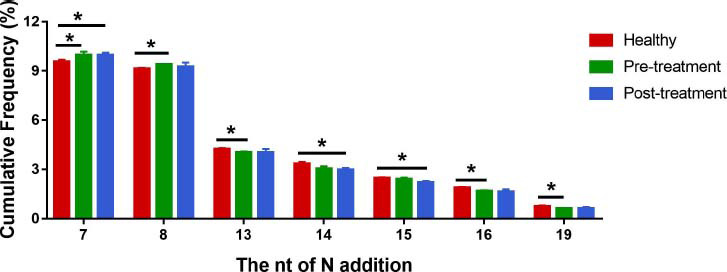
Nucleotide addition frequency in samples of the three groups. Frequency of nucleotide addition in subjects from the healthy control group (red), pretreatment group (green), and posttreatment group (blue), showing only significant differences. *P < 0.05.

Compared to healthy controls, KOA patients showed a significant increase in addition frequency for 7 and 8 nucleotides (*P <* 0.05) and a significant decrease in addition frequency for 13, 14, 15, 16, and 19 nucleotides (*P <* 0.05). Compared to pretreatment data, KOA patients post EA showed a decreasing tendency in addition frequency for 7 and 8 nucleotides and an increasing tendency for 13, 14, 15, 16, and 19 nucleotides, although there was no significant difference (Fig. **7**).

#### 3.2.7 Percentage of CDR3 with Different Read Numbers in Total CDR3

**Fig. (8):**
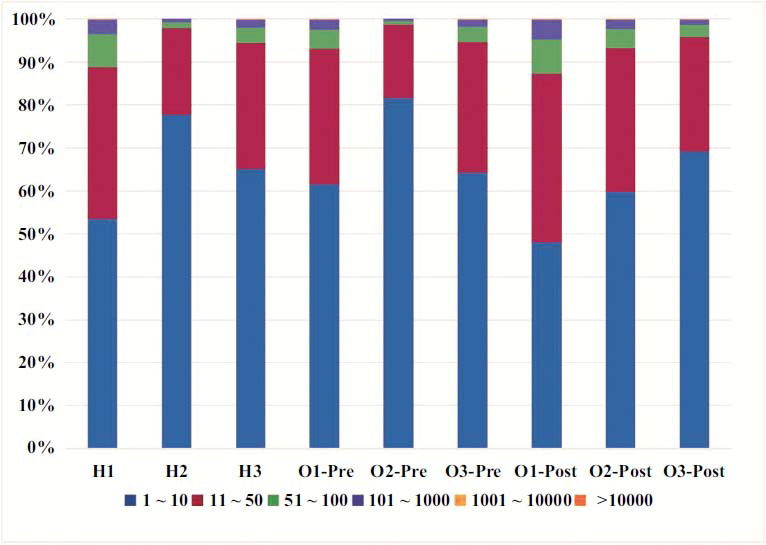
The percentage of CDR3 with different read numbers in total CDR3. H1, H2, H3: Healthy control subjects; O1-Pre, O2-Pre, O3-Pre: KOA subjects prior to treatment; O1-Post, O2-Post, O3-Post: KOA subjects post to treatment.

CDR3 was categorized into 6 groups based on sequencing abundance. The read number of CDR3 was categorized into 6 groups based on ascending order: 1-10, 11-50, 51-100, 101-1000 and >1000. The distribution of CDR3 frequency was compared among these 6 groups. CDR3 in all samples was dominated by read numbers of 1-100, without any significant difference in the percentage of CDR3 with different read numbers in total CDR3 among the groups. These results suggest that there was no statistically significant difference in CDR3 with various read numbers in samples among all groups (Fig. **8**).

#### 3.2.8 Tree Map of TCR Diversity, 2D and 3D Map of V-J Gene Combinations

**Fig. (9):**
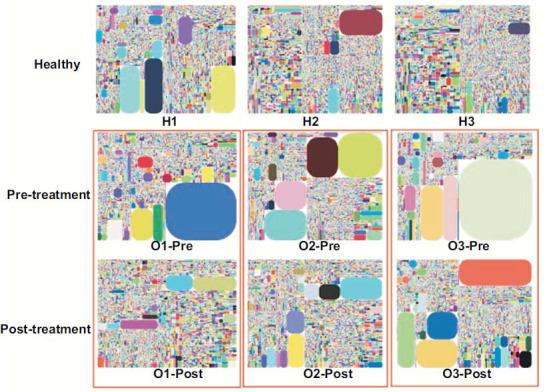
Tree map of TCRβ CDR3 from all samples. TCR repertoire diversity is illustrated in a tree map where each rounded rectangle represents a unique entry: V-J-uCDR3 and the size of the spot denotes the relative frequency. H1, H2, H3: Healthy control subjects; O1-Pre, O2-Pre, O3-Pre: KOA subjects prior to treatment; O1-Post, O2-Post, O3-Post: KOA subjects post to treatment.

The treemap showed that KOA patients prior to treatment exhibited higher levels of large clonal CDR3 with a large sequence number than healthy controls. The 2D heat map and 3D map also revealed that the identified combination of V- and J-gene segments was obviously lower in KOA subjects prior to treatment than in healthy controls. The treemap yielded results indicating a decreasing sequence number of large clonal CDR3 in KOA patients posttreatment, with an obvious increase in IR diversity (Fig. **9**; Supplementary Fig. **1**, **2**).

### 3.3 Serum Inflammatory Cytokines

Compared to pretreatment levels, posttreatment KOA patients showed a significant decrease in serum proinflammatory cytokines IL-8 and IL-18 (*P <* 0.01, both). There were no significant differences in IL-1β, CC chemokine ligand-5 (CCL-5), TNF-α or monocyte chemotactic protein 1 (MCP-1), but all showed a decreasing trend (Supplementary Fig. **3**). The anti-inflammatory cytokines IL-10 and IL-13 were significantly higher posttreatment than pretreatment (*P* < 0.01, both); there was no significant difference in IL-4, but it showed an increasing trend (Supplementary Fig. **4**).

### 3.4 Serum Matrix Metalloproteinase

Compared to pretreatment levels, posttreatment KOA patients showed a significant reduction in serum matrix metalloproteinase-3 and -13 (MMP-3 and -13) (*P <* 0.01, *P <* 0.05) (Supplementary Fig. **5**).

### 3.5 Correlation Analysis of Serum Inflammatory Cytokines, MMPs and TCR Repertoire

**Fig. (10):**
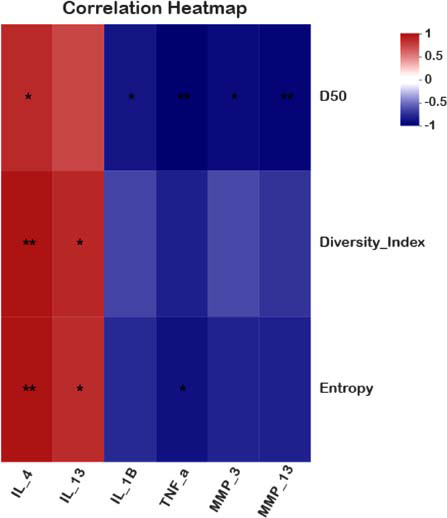
Correlation analysis of serum inflammatory cytokines, MMPs and TCR repertoire. Red represents a positive correlation, and blue represents a negative correlation. The depth of the color represents the strength of the correlation between the two factors.

Correlative assessments were made on the inflammatory cytokines, MMPs and TCR repertoire. The results showed that DI, D50, and entropy were positively correlated with the anti-inflammatory cytokine IL-4; DI and entropy were positively correlated with the anti-inflammatory cytokine IL-13. D50 was negatively correlated with the proinflammatory cytokines IL-1β and TNF-α and cartilage degradation biomarkers MMP-3 and MMP-13 (Fig. **10**).

### 3.6 Transcriptome Sequencing

**Fig. (11):**
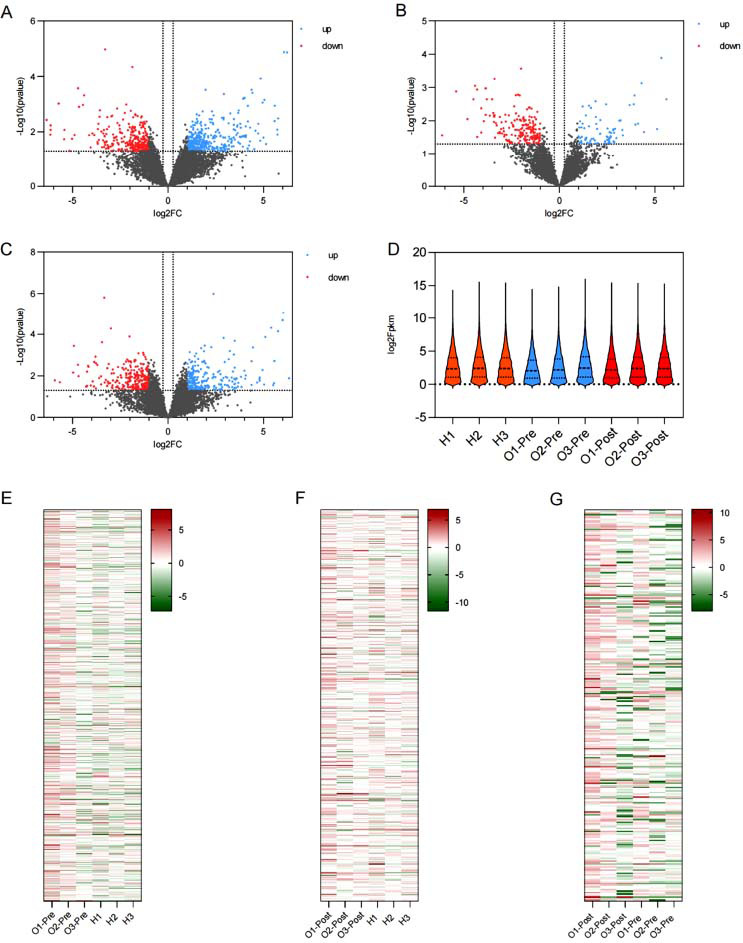
Transcriptomics sequencing of pretreatment patients *versus* posttreatment patients for a healthy population. (**A**) Volcano plot of pretreatment patients and healthy population. (**B**) Volcano plot of posttreatment patients *vs*. pretreatment patients. (**C**) Volcano plot of posttreatment patients *versus* healthy population. (**D**) A healthy population, violin plot of pretreatment patients *versus* posttreatment patients. (**E**) Heat map of pretreatment patients and healthy population. (**F**) Heat map of posttreatment patients *versus* pretreatment patients. (**G**) Heat map of posttreatment patients *versus* healthy population.

**Fig. (12):**
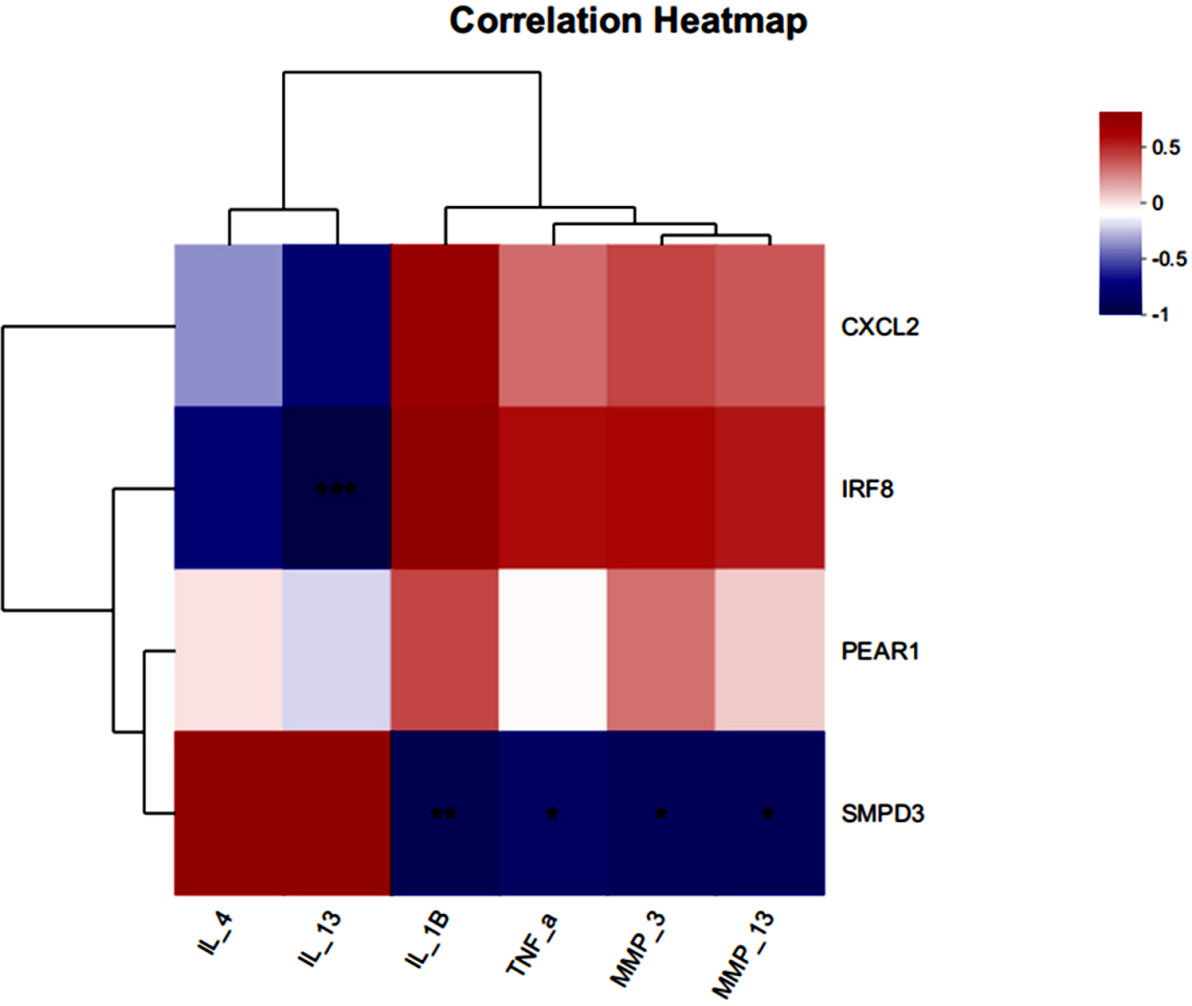
Correlation analysis of serum inflammatory cytokines, MMPs and key genes.

**Fig. (13):**
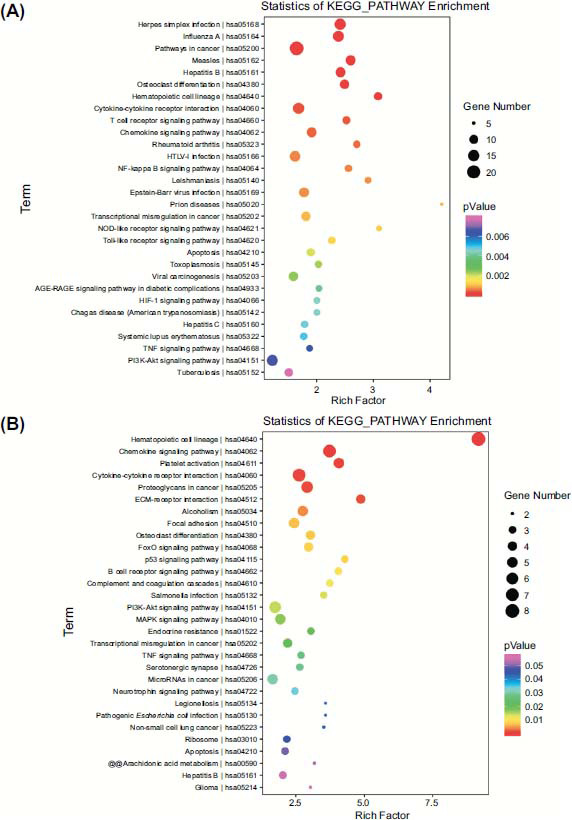
KEGG metabolic pathway enrichment map. (**A**) Enrichment maps of KEGG pathways in pretreatment patients and healthy populations. (**B**) Enrichment maps of KEGG pathways in posttreatment patients *versus* pretreatment patients.

We sequenced the neutrophil mRNA transcriptomes of pre/post-treatment patients and the healthy population, of which 810 genes were differentiated between the pretreatment and healthy populations, of which 395 genes were upregulated, and 294 genes were downregulated, and the heatmap also showed a significant difference between the two groups (Fig. **11A**-**E**). 70 genes were upregulated in the posttreatment population *versus* the pretreatment population, and 263 genes were downregulated (Fig. **11B** and **10G**). Two hundred fifty-six genes were upregulated, and 263 genes were downregulated in the posttreatment population versus the healthy population (Fig. **11C**-**F**). We performed the intersection of the differential genes produced by the pretreatment patients compared to the healthy population and the differential genes produced by the posttreatment patients compared to the pretreatment patients, and 72 of these genes did not differ between the posttreatment population and the healthy population, demonstrating that these 72 genes did not differ from the healthy population when they were significantly improved by treatment (Supplementary Table **4**, **5**; Supplementary Fig. **6**). CXCL2, IRF8, SMPD3, and PEAR1 attracted our attention, and all of them were significantly altered by the treatment (Supplementary Tables **4** and **5**). Correlative assessments were made on the inflammatory cytokines and the key genes. The results showed that IRF8 was negatively correlated with the anti-inflammatory cytokine IL-13; SMPD3 was negatively correlated with the pro-inflammatory cytokines IL-1β and TNF-α and cartilage degradation biomarkers MMP-3 and MMP-13 (Fig. **12**). Metabolic pathways associated with KOA cartilage destruction: osteoclast differentiation, and metabolic pathways associated with chondrocyte injury: apoptosis, are significantly enriched in healthy populations *versus* pre-treatment patients. T cell receptor signaling pathway, Chemokine signaling pathway, TNF signaling pathway, PI3K-Akt signaling pathway and inflammatory factor-related metabolic pathways were significantly enriched in pre-treatment patients *versus* the healthy population (Fig. **13A**). After treatment, we found that immune-related pathways such as the Chemokine signaling pathway, Cytokine-cytokine receptor interaction, and Focal adhesion were significantly improved (Fig. **13B**).

## 4 DISCUSSION

IR reflects the systemic immune system's potential to respond to external stimuli and can be used to evaluate overall immune function. TCR monitors the immune microenvironment and regulates immunity. The initiation of adaptive immunity depends on TCR diversity, and current studies are exploring the value of the TCR database [25-29]. TCR consists of two chains (α and β) linked by a disulfide bond, with three hypervariable regions (CDR1, CDR2, CDR3) in the β chain's variable region. The highly variable CDR3 loop is produced by recombination of V, D, and J gene fragments, along with nucleotide deletion and insertion at the V(D)J junction. The diversity of the CDR3 sequence, due to its close interaction with antigen peptides, measures T-cell diversity [29, 30]. Systemic immunity can be assessed by the CDR3 diversity of TCRs in peripheral blood. Evidence shows that diseases like myocardial infarction and malignancies are associated with a significant decrease in CDR3 diversity, correlating with patient prognosis [31-33]. Jin-Huan Cui sequenced the β chain of TCR in peripheral blood in patients with cervical cancer or cervical intraepithelial neoplasia and healthy women [33]. The results showed a decrease in TCR clone types and a significant reduction in TCR diversity in patients with cervical cancer compared to the other two groups [33]. Additionally, IR diversity gradually decreases with age. Research on the TCR β chain in healthy volunteers aged 6 to 90 showed a roughly linear decline in TCR diversity, with a noticeable drop after age 40 [34].

This study revealed a significant decrease in CDR3 diversity of TCRs in KOA patients compared to healthy controls. Previous studies emphasize the role of immunity-mediated T cells in KOA pathogenesis [34, 35]. T cells and effector cells in the adaptive immune response play roles through proliferation, production of cytokines, cytotoxicity and differentiation [36, 37]. Although an increase in CD8+ T cells correlates with aging in KOA patients, other research indicates that T cell-driven inflammation in KOA may alter cellular functions beyond the CD4+/CD8+ imbalance [18]. Immune aging, defined as a decline in immune function correlated with aging, is complicated by low-grade inflammation in the host [35]. Aging and other factors may cause CD4+ T cells in KOA patients to become hypofunctional and dominated by dysfunctional phenotypes. Age should be considered in analyses of T-cell immune function. In this study, healthy controls were matched with KOA subjects by age and sex [38]. All healthy volunteers underwent physical examinations before inclusion. Subjects with chronic or major diseases, or those on medication, were excluded. Bilateral knee X-rays were performed to rule out KOA [39]. Therefore, age, sex, diseases, or medication did not influence the differences in peripheral T cell IR results between the groups. This study showed a significant decrease in D50, DI of CDR3, and Shannon entropy in circulatory T cells of KOA patients compared to healthy controls (*P* < 0.05) (Fig. **3**). The treemap results showed increased large clonal CDR3 sequences in KOA patients before treatment compared to healthy controls, indicating reduced IR diversity and the emergence of large clonal CDR3 in KOA (Fig. **9**). These findings, along with previous studies, suggest that a decrease in T cell-mediated adaptive immunity and the expansion of specific CDR3 clones are pathogenic factors in KOA.

Although not statistically significant, there was a decreasing trend in the mean WOMAC pain, stiffness, joint function, total scores, and NRS in KOA patients after 8 weeks of EA compared to pre-intervention data. This may be due to the small sample size. Studies indicate that treatment response in KOA patients can be confirmed by achieving minimal clinically important improvement (MCII), defined as a decrease of 2 points in the NRS and 6 points in the WOMAC subscales from baseline [40, 41]. The reduction from 7.7 to 3.7 and from 14.0 to 6.3 for the mean NRS and mean WOMAC subscale, respectively, as shown by this study, seems consistent with MCII for KOA and confirms the response in KOA patients to EA (Supplement Table **2**).

TCR clonotype and diversity are key aspects of immunity. KOA patients showed a significant increase in TCR DI and Shannon entropy post-EA compared to pretreatment (both *P* < 0.01) (Supplementary Table **3**). The treemap revealed a decrease in large clonal CDR3 sequences and an increase in IR diversity post-EA, indicating the intervention's effectiveness in improving T-cell receptor diversity in KOA patients (Fig. **9**). This effect is likely due to enhanced T-cell IR diversity and reduced large clonal status of pathogenic CDR3.

Research has placed a high value on inflammatory factors in the pathophysiology of KOA. [40]. The elevation of IL-1, IL-18, and TNF-α in tissue and IL-8 content in synovial fluid is involved in the pathogenesis of KOA [42-45]. The level of MMP13 in the serum of KOA patients is negatively correlated with knee cartilage volume and positively correlated with the levels of serum TNF-a, IL-8, and IL-18 as well as cartilage defects, suggesting the potential efficacy of circulatory MMP13 in the pathogenesis of KOA and the possible regulation by inflammatory factors upon MMP13 [46, 47]. As an important effector in cartilage metabolism, MMP-3 also plays a vital role in the pathogenic mechanism of KOA, and its level is related to the reduction of cartilage volume [25, 48, 49]. Our preliminary study showed that eight weeks of EA or manual acupuncture effectively relieved knee pain and improved knee function in patients with mild to moderate KOA. It significantly lowered levels of proinflammatory cytokines TNF-α, IL-1β, MMP-3, and MMP-13 (markers of cartilage degradation) and increased anti-inflammatory IL-13 levels. In this study, post-treatment KOA patients showed significant decreases in serum IL-8 and IL-18 (both *P* < 0.01), a trend toward lower IL-1β and TNF-α, substantial increases in anti-inflammatory IL-10 and IL-13 *(P* < 0.01, *P* < 0.05), and significant reductions in MMP-3 and MMP-13 (both *P* < 0.01), consistent with previous research. Thus, EA's efficacy in treating KOA may be achieved by reducing proinflammatory factors and MMPs while increasing anti-inflammatory factors (Supplementary Figs. **4-6**).

Studies have confirmed that host immune status determines the outcome of inflammatory reactions to a large extent [50, 51]. TCR diversity reflects systemic immunity, but prolonged inflammation inhibits T lymphocyte activation and induces low T cell reactivity. Thitiya and colleagues analyzed the immune repertoire of activated CD8+ T cells, comparing T cell receptor β-variable gene chain (TRBV) usage in peripheral blood and infrapatellar fat pads (IPFP) of knee OA patients [52]. They found that activated CD8^+^ IPFP T cells carry different repertoire distribution from those in the peripheral blood of KOA patients, and shared TRBV usage of activated CD8^+^ IPFP T cells among the 3 patients with knee OA was also identified [53]. However, it remains unknown if the immune repertoire differs between KOA patients and healthy volunteers or if it changes after EA treatment. Our study found that KOA patients' immune repertoire differed from healthy volunteers and significantly changed after 8 weeks of EA treatment [54, 55]. Furthermore, the shared TRBV4-6, TRBV20-1, and TRBV28 identified in the peripheral blood of three KOA patients in Thitiya’s study were also detected in our study (Supplementary Fig. **1**). These results suggested that immune repertoire sequencing is a useful and reliable method for KOA study.

There is a mutual influence between inflammation and immunity. In KOA patients, anti-inflammatory factors IL-4 and IL-13 positively correlated with DI and entropy, while proinflammatory factors IL-1β and TNF-α and cartilage degradation markers MMP-13 and MMP-3 negatively correlated with D50 (Fig. **10**). Eight weeks of EA improved clinical symptoms, increased circulating IL-13, reduced MMP-13 and MMP-3, and significantly promoted TCR diversity. These findings support the effectiveness of EA in regulating systemic inflammation and immunity in KOA patients. EA likely modulates inflammatory factor release by enhancing TCR diversity, thereby reducing proinflammatory factors, increasing anti-inflammatory factors, lowering MMP production, and protecting knee cartilage, ultimately achieving its therapeutic effect against KOA.

The development of osteoarthritis is closely linked to immune abnormalities, with various immune cells and mediators playing critical roles in its pathogenesis [10, 53]. CXCL2, also known as macrophage inflammatory protein-2, is a chemokine that attracts immune cells like neutrophils, monocytes, and lymphocytes to inflammatory sites or damaged tissues, regulating inflammation and immune responses [56]. It promotes inflammation by stimulating cells to release mediators such as TNF-α, IL-1β, and IL-8, exacerbating the inflammatory process [10, 57, 58]. CXCL2 also enhances the antimicrobial capacity of immune cells, participates in cell interactions, and regulates processes like apoptosis and proliferation [59]. In this study, CXCL2 was significantly upregulated in KOA patients before treatment compared to healthy controls but significantly downregulated after EA treatment, aligning with levels in the healthy population. This suggests that CXCL2 is overexpressed in KOA, exacerbating inflammation, and EA effectively reduces its expression (Supplementary Tables **4** and **5**).

IRF8 is one of the important factors which could regulate the immune response. It promotes macrophage function, including phagocytosis and the killing of pathogens such as bacteria and viruses [60]. IRF8 also cloud encourage presentation antigen of T cells and the activation functions of dendritic cells [60, 61]. The expression of IRF8 can be regulated by a variety of immune stimuli. Of these, the most important are interferon and Toll-like receptor (TLR) activation [62]. Interferon is an important immunomodulatory factor that activates IRF8 expression, thereby promoting the immune function of macrophages and dendritic cells [10]. Activation of TLR, a receptor that recognizes pathogen molecules, leads to an upregulation of IRF8 expression, thereby promoting an immune response [10]. The results showed that IRF8 was significantly upregulated in the pretreatment patients compared to the healthy population and decreased significantly after EA treatment. Moreover, there was no difference between the healthy population and the posttreatment patients (Supplementary Table **4** and **5**).

The PEAR1 gene is related to immune response. It encodes a protein that binds to TLRs, triggering an inflammatory response. Overexpression of the PEAR1 gene promotes inflammation, leading to tissue damage and inflammatory diseases [63, 64]. Changes in PEAR1 gene expression levels can affect the intensity and duration of the inflammatory response [65]. Excessive PEAR1 expression can lead to uncontrolled inflammation, worsening inflammatory disease symptoms. Conversely, reduced PEAR1 expression can limit inflammation and alleviate symptoms [66, 67]. Our results showed that PEAR1 was upregulated in patients before treatment but significantly decreased after EA treatment, suggesting that EA may improve the inflammatory response (Supplementary Tables **4** and **5**).

The SMPD3 gene likely plays a key role in biological processes such as the cell cycle, organ development, exosome secretion, and sphingolipid metabolism. These processes may be indirectly related to cartilage damage [68, 69]. For example, exosome secretion and sphingolipid metabolism processes may be associated with chondrocyte growth, differentiation, and repair [70]. If these processes are disturbed or abnormal, they can adversely affect cartilage. Smpd3 expression in both chondrocytes and osteoblasts is required for normal endochondral bone development [71]. SMPD3 deficiency is the pathogenetic basis of a novel form of chondrodysplasia [72]. The dysfunction of SMPD3 enhanced the TLR-induced inflammatory response of B cells and macrophages in turn [73]. Our study found that the expression of the SMPD3 gene was downregulated in patients prior to treatment, and there was significant upregulation after treatment, suggesting that EA could promote chondrocyte development (Supplementary Table **4** and **5**).

Previous studies have linked osteoarthritis onset to cartilage wear and tear. Recent research suggests it is a chronic inflammatory state involving extensive immune cell activity. In-depth studies on osteoarthritis pathogenesis indicate that early innate immune responses drive the disease, gradually causing degenerative changes and altering the joint microenvironment [74, 75]. Various immune cells and cytokines are key factors in osteoarthritis repair. Early evaluation of immunological risk factors can enable effective treatment, significantly reducing disability, morbidity, and costs associated with KOA [10]. Our study showed significant enrichment of cytokine-cytokine receptor interaction, TNF signaling pathway, and PI3K-Akt signaling pathway in pretreatment *versus* healthy populations and posttreatment *versus* pretreatment populations (Fig. **13**). Correlation analyses revealed that IRF8 was significantly negatively correlated with IL-13, and AMPD3 was significantly negatively correlated with IL-1β, TNFα, MMP-3, and MMP-13 (Fig. **12**).

## CONCLUSION

KOA patients experienced improvements in pain, stiffness, and joint function after 8 weeks of EA treatment. There was a significant increase in TCR diversity and anti-inflammatory cytokines IL-10 and IL-13 in peripheral blood, while proinflammatory cytokines IL-8 and IL-18, as well as cartilage degradation biomarkers MMP-3 and MMP-13, were significantly decreased. Transcriptome sequencing showed overexpression of IRF8, PEAR1, and CXCL2 in KOA patients, exacerbating inflammation. However, these expressions significantly decreased after EA treatment. EA may regulate the release of inflammatory factors by modulating immune cell structure and function, reducing pathogenic biomarkers, and mitigating knee tissue damage. The significant upregulation of SMPD3 gene expression suggests that EA may promote chondrocyte development. The small sample size of the study and focus on differences between pretreatment (week 0) and posttreatment (week 8) periods indicate that further studies with larger sample sizes and longer-term observations are needed.

## Data Availability

The authors confirm that the data supporting the findings of this research are available within the article.

## LIST OF ABBREVIATIONS

CCL-5: CC Chemokine Ligand-5
CD8+: Cluster of Differentiation 8
CDR3: Complementarity Determining Region 3
CXCL2: C-X-C Motif Chemokine Ligand 2
DI: Diversity Index
EA: Electroacupuncture
ELISA: Enzyme-Linked Immunosorbent Assay
IL-8: Interleukin-8
IPFP: Infrapatellar Fat Pad
IR: Immune Repertoire
IRF8: Interferon Regulatory Factor 8
MCP-1: Monocyte Chemotactic Protein-1
MMP-3: Metalloproteinase-3
NF-κB: Nuclear Factor Kappa-Light-Chain-Enhancer of Activated B Cells
NRS: Numerical Rating Scale
PEAR1: Platelet Endothelial Cell Adhesion Molecule-1
PI3K-Akt: Phosphatidylinositol 3-Kinase-Akt Signaling Pathway
RIN: RNA Integrity Number
SMPD3: Sphingomyelin Phosphodiesterase 3
TLR: Toll-Like Receptor
TNF-α: Tumor Necrosis Factor-alpha
TRBV: T Cell Receptor β-Variable Gene
WOMAC: Western Ontario and McMaster Universities Osteoarthritis Index

## STANDARDS OF REPORTING

CONSORT guidelines were followed.

## References

[1] Hochberg M.C., Altman R.D., April K.T., Benkhalti M., Guyatt G., McGowan J., Towheed T., Welch V., Wells G., Tugwell P. (2012). American college of rheumatology 2012 recommendations for the use of nonpharmacologic and pharmacologic therapies in osteoarthritis of the hand, hip, and knee.. Arthritis Care Res..

[2] Pereira D., Peleteiro B., Araújo J., Branco J., Santos R.A., Ramos E. (2011). The effect of osteoarthritis definition on prevalence and incidence estimates: A systematic review.. Osteoarthritis Cartilage.

[3] Vincent H.K., Percival S.S., Conrad B.P., Seay A.N., Montero C., Vincent K.R. (2013). Hyaluronic acid (HA) viscosupplementation on synovial fluid inflammation in knee osteoarthritis: A pilot study.. Open Orthop. J..

[4] Livshits G., Zhai G., Hart D.J., Kato B.S., Wang H., Williams F.M.K., Spector T.D. (2009). Interleukin‐6 is a significant predictor of radiographic knee osteoarthritis: The Chingford study.. Arthritis Rheum..

[5] Orlowsky E.W., Kraus V.B. (2015). The role of innate immunity in osteoarthritis: When our first line of defense goes on the offensive.. J. Rheumatol..

[6] Sae-jung T., Sengprasert P., Apinun J., Ngarmukos S., Yuktanandana P., Tanavalee A., Reantragoon R. (2019). Functional and T cell receptor repertoire analyses of peripheral blood and infrapatellar fat pad T cells in knee osteoarthritis.. J. Rheumatol..

[7] Toncheva A., Remichkova M., Ikonomova K., Dimitrova P., Ivanovska N. (2009). Inflammatory response in patients with active and inactive osteoarthritis.. Rheumatol. Int..

[8] Filková M., Lišková M., Hulejová H., Haluzík M., Gatterová J., Pavelková A., Pavelka K., Gay S., Müller-Ladner U., Šenolt L. (2009). Increased serum adiponectin levels in female patients with erosive compared with non-erosive osteoarthritis: Figure 1.. Ann. Rheum. Dis..

[9] Perruccio A.V., Mahomed N.N., Chandran V., Gandhi R. (2014). Plasma adipokine levels and their association with overall burden of painful joints among individuals with hip and knee osteoarthritis.. J. Rheumatol..

[10] Beekhuizen M., Gierman L.M., van Spil W.E., Van Osch G.J.V.M., Huizinga T.W.J., Saris D.B.F., Creemers L.B., Zuurmond A.M. (2013). An explorative study comparing levels of soluble mediators in control and osteoarthritic synovial fluid.. Osteoarthritis Cartilage.

[11] (2024). Retraction: MicroRNA-26a reduces synovial inflammation and cartilage injury in osteoarthritis of knee joints through impairing the NF-κB signaling pathway.. Biosci. Rep..

[12] Wilhelmsen K., Farrar K., Hellman J. (2013). Quantitative *in vitro* assay to measure neutrophil adhesion to activated primary human microvascular endothelial cells under static conditions.. J. Vis. Exp..

[13] Jaeschke H. (2006). Mechanisms of liver injury. II. Mechanisms of neutrophil-induced liver cell injury during hepatic ischemia-reperfusion and other acute inflammatory conditions.. Am. J. Physiol. Gastrointest. Liver Physiol..

[14] Jordan K.M., Arden N.K., Doherty M., Bannwarth B., Bijlsma J.W., Dieppe P., Gunther K., Hauselmann H., Herrero-Beaumont G., Kaklamanis P., Lohmander S., Leeb B., Lequesne M., Mazieres B., Martin-Mola E., Pavelka K., Pendleton A., Punzi L., Serni U., Swoboda B., Verbruggen G., Zimmerman-Gorska I., Dougados M. (2003). EULAR recommendations 2003: An evidence based approach to the management of knee osteoarthritis: Report of a task force of the standing committee for international clinical studies including therapeutic trials (ESCISIT).. Ann. Rheum. Dis..

[15] (2014). National Clinical Guideline Centre (UK). Osteoarthritis: Care and Management in Adults.

[16] Corbett M.S., Rice S.J.C., Madurasinghe V., Slack R., Fayter D.A., Harden M., Sutton A.J., MacPherson H., Woolacott N.F. (2013). Acupuncture and other physical treatments for the relief of pain due to osteoarthritis of the knee: Network meta-analysis.. Osteoarthritis Cartilage.

[17] Shi G.X., Tu J.F., Wang T.Q., Yang J.W., Wang L.Q., Lin L.L., Wang Y., Li Y.T., Liu C.Z. (2020). Effect of electro-acupuncture (EA) and manual acupuncture (MA) on markers of inflammation in knee osteoarthritis.. J. Pain Res..

[18] Torres-Rosas R., Yehia G., Peña G., Mishra P., del Rocio Thompson-Bonilla M., Moreno-Eutimio M.A., Arriaga-Pizano L.A., Isibasi A., Ulloa L. (2014). Dopamine mediates vagal modulation of the immune system by electroacupuncture.. Nat. Med..

[19] Friedensohn S., Khan T.A., Reddy S.T. (2017). Advanced methodologies in high-throughput sequencing of immune repertoires.. Trends Biotechnol..

[20] Liu X., Wu J. (2018). History, applications, and challenges of immune repertoire research.. Cell Biol. Toxicol..

[21] Tu J.F., Yang J.W., Shi G.X., Yu Z.S., Li J.L., Lin L.L., Du Y.Z., Yu X.G., Hu H., Liu Z.S., Jia C.S., Wang L.Q., Zhao J.J., Wang J., Wang T., Wang Y., Wang T.Q., Zhang N., Zou X., Wang Y., Shao J.K., Liu C.Z. (2021). Efficacy of intensive acupuncture versus sham acupuncture in knee osteoarthritis: A randomized controlled trial.. Arthritis Rheumatol..

[22] Peat G., Thomas E., Duncan R., Wood L., Hay E., Croft P. (2006). Clinical classification criteria for knee osteoarthritis: Performance in the general population and primary care.. Ann. Rheum. Dis..

[23] Wu C.W., Morrell M.R., Heinze E., Concoff A.L., Wollaston S.J., Arnold E.L., Singh R., Charles C., Skovrun M.L., FitzGerald J.D., Moreland L.W., Kalunian K.C. (2005). Validation of american college of rheumatology classification criteria for knee osteoarthritis using arthroscopically defined cartilage damage scores.. Semin. Arthritis Rheum..

[24] Salaffi F., Leardini G., Canesi B., Mannoni A., Fioravanti A., Caporali R., Lapadula G., Punzi L. (2003). Reliability and validity of the western ontario and mcmaster universities (WOMAC) osteoarthritis index in italian patients with osteoarthritis of the knee.. Osteoarthritis Cartilage.

[25] Emerson R.O., Sherwood A.M., Rieder M.J., Guenthoer J., Williamson D.W., Carlson C.S., Drescher C.W., Tewari M., Bielas J.H., Robins H.S. (2013). High‐throughput sequencing of T‐cell receptors reveals a homogeneous repertoire of tumour‐infiltrating lymphocytes in ovarian cancer.. J. Pathol..

[26] Liu X., Zhang W., Zhao M., Fu L., Liu L., Wu J., Luo S., Wang L., Wang Z., Lin L., Liu Y., Wang S., Yang Y., Luo L., Jiang J., Wang X., Tan Y., Li T., Zhu B., Zhao Y., Gao X., Wan Z., Huang C., Fang M., Li Q., Peng H., Liao X., Chen J., Li F., Ling G., Zhao H., Luo H., Xiang Z., Liao J., Liu Y., Yin H., Long H., Wu H. (2019). Yang; Wang, J.; Lu, Q. T cell receptor β repertoires as novel diagnostic markers for systemic lupus erythematosus and rheumatoid arthritis.. Ann. Rheum. Dis..

[27] Li Y., Wang X., Teng D., Chen H., Wang M., Wang J., Zhang J., He W. (2019). Identification of the ligands of TCRγδ by screening the immune repertoire of γδT cells from patients with tuberculosis.. Front. Immunol..

[28] Lin Z., Qian S., Gong Y., Ren J., Zhao L., Wang D., Wang X., Zhang Y., Wang Z., Zhang Q. (2017). Deep sequencing of the T cell receptor β repertoire reveals signature patterns and clonal drift in atherosclerotic plaques and patients.. Oncotarget.

[29] Liu H., Pan W., Tang C., Tang Y., Wu H., Yoshimura A., Deng Y., He N., Li S. (2021). The methods and advances of adaptive immune receptors repertoire sequencing.. Theranostics.

[30] Yao X.S., Diao Y., Sun W.B., Luo J.M., Qin M., Tang X.Y. (2007). Analysis of the CDR3 length repertoire and the diversity of TCR alpha chain in human peripheral blood T lymphocytes.. Cell. Mol. Immunol..

[31] Li D., Hu L., Liang Q., Zhang C., Shi Y., Wang B., Wang K. (2019). Peripheral T cell receptor beta immune repertoire is promptly reconstituted after acute myocardial infarction.. J. Transl. Med..

[32] Zhong Z., Wu H., Zhang Q., Zhong W., Zhao P. (2019). Characteristics of T cell receptor repertoires of patients with acute myocardial infarction through high-throughput sequencing.. J. Transl. Med..

[33] Cui J.H., Lin K.R., Yuan S.H., Jin Y.B., Chen X.P., Su X.K., Jiang J., Pan Y.M., Mao S.L., Mao X.F., Luo W. (2018). TCR repertoire as a novel indicator for immune monitoring and prognosis assessment of patients with cervical cancer.. Front. Immunol..

[34] Britanova O.V., Putintseva E.V., Shugay M., Merzlyak E.M., Turchaninova M.A., Staroverov D.B., Bolotin D.A., Lukyanov S., Bogdanova E.A., Mamedov I.Z., Lebedev Y.B., Chudakov D.M. (2014). Age-related decrease in TCR repertoire diversity measured with deep and normalized sequence profiling.. J. Immunol..

[35] Sadighi Akha A.A. (2018). Aging and the immune system: An overview.. J. Immunol. Methods.

[36] Manjunath N., Shankar P., Wan J., Weninger W., Crowley M.A., Hieshima K., Springer T.A., Fan X., Shen H., Lieberman J., von Andrian U.H. (2001). Effector differentiation is not prerequisite for generation of memory cytotoxic T lymphocytes.. J. Clin. Invest..

[37] Xiao J., Zhang P., Cai F.L., Luo C.G., Pu T., Pan X.L., Tian M. (2023). IL-17 in osteoarthritis: A narrative review.. Open Life Sci..

[38] Sotiropoulos C., Theodorou G., Repa C., Marinakis T., Verigou E., Solomou E., Karakantza M., Symeonidis A. (2014). Severe impairment of regulatory T-cells and Th1-lymphocyte polarization in patients with Gaucher disease.. JIMD Rep..

[39] Yamashina S., Harada K., Tanaka R., Inoue Y. (2023). Abnormal gait pattern examination screening for physical activity level after one year in patients with knee osteoarthritis.. J. Funct. Morphol. Kinesiol..

[40] Tubach F., Ravaud P., Baron G., Falissard B., Logeart I., Bellamy N., Bombardier C., Felson D., Hochberg M., van der Heijde D., Dougados M. (2005). Evaluation of clinically relevant changes in patient reported outcomes in knee and hip osteoarthritis: The minimal clinically important improvement.. Ann. Rheum. Dis..

[41] Ornetti P., Dougados M., Paternotte S., Logeart I., Gossec L. (2011). Validation of a numerical rating scale to assess functional impairment in hip and knee osteoarthritis: Comparison with the WOMAC function scale.. Ann. Rheum. Dis..

[42] Kapoor M., Martel-Pelletier J., Lajeunesse D., Pelletier J.P., Fahmi H. (2011). Role of proinflammatory cytokines in the pathophysiology of osteoarthritis.. Nat. Rev. Rheumatol..

[43] Wang Y., Xu D., Long L., Deng X., Tao R., Huang G. (2014). Correlation between plasma, synovial fluid and articular cartilage Interleukin-18 with radiographic severity in 33 patients with osteoarthritis of the knee.. Clin. Exp. Med..

[44] Kaneko S., Satoh T., Chiba J., Ju C., Inoue K., Kagawa J. (2000). Interleukin–6 and interleukin–8 levels in serum and synovial fluid of patients with osteoarthritis.. Cytokines Cell. Mol. Ther..

[45] Monibi F., Roller B., Stoker A., Garner B., Bal S., Cook J. (2015). Identification of synovial fluid biomarkers for knee osteoarthritis and correlation with radiographic assessment.. J. Knee Surg..

[46] Ruan G., Xu J., Wang K., Wu J., Zhu Q., Ren J., Bian F., Chang B., Bai X., Han W., Ding C. (2018). Associations between knee structural measures, circulating inflammatory factors and MMP13 in patients with knee osteoarthritis.. Osteoarthritis Cartilage.

[47] Wang K., Xu J., Cai J., Zheng S., Yang X., Ding C. (2017). Serum levels of resistin and interleukin-17 are associated with increased cartilage defects and bone marrow lesions in patients with knee osteoarthritis.. Mod. Rheumatol..

[48] Du F., Lü L., Teng J., Shen N., Ye P., Bao C. (2012). T-614 alters the production of matrix metalloproteinases (MMP-1 andMMP-3) and inhibits the migratory expansion of rheumatoid synovial fibroblasts, in vitro.. Int. Immunopharmacol..

[49] Wojdasiewicz P., Poniatowski Ł.A., Szukiewicz D. (2014). The role of inflammatory and anti-inflammatory cytokines in the pathogenesis of osteoarthritis.. Mediators Inflamm..

[50] Hensley M., Deng J. (2018). Acute on chronic liver failure and immune dysfunction: A mimic of sepsis.. Semin. Respir. Crit. Care Med..

[51] Shankar Hari M., Summers C. (2018). Major surgery and the immune system: From pathophysiology to treatment.. Curr. Opin. Crit. Care.

[52] Ramesh M., Hamm D., Simchoni N., Cunningham-Rundles C. (2017). Clonal and constricted T cell repertoire in common variable immune deficiency.. Clin. Immunol..

[53] Apinun J., Sengprasert P., Yuktanandana P., Ngarmukos S., Tanavalee A., Reantragoon R. (2016). Immune mediators in osteoarthritis: Infrapatellar fat pad-infiltrating CD8+ T cells are increased in osteoarthritic patients with higher clinical radiographic grading.. Int. J. Rheumatol..

[54] Hopkins A.C., Yarchoan M., Durham J.N., Yusko E.C., Rytlewski J.A., Robins H.S., Laheru D.A., Le D.T., Lutz E.R., Jaffee E.M. (2018). T cell receptor repertoire features associated with survival in immunotherapy-treated pancreatic ductal adenocarcinoma.. JCI Insight.

[55] Wang T.Q., Li L.R., Tan C.X., Yang J.W., Shi G.X., Wang L.Q., Hu H., Liu Z.S., Wang J., Wang T., Yuan Y., Jia W.R., Li H., Wang X.W., Wu B., Tu J.F., Liu C.Z. (2021). Effect of electroacupuncture on gut microbiota in participants with knee osteoarthritis.. Front. Cell. Infect. Microbiol..

[56] Gu Y., Zhang X., Li H., Wang R., Jin C., Wang J., Jin Z., Lu J., Ling C., Shao F., Zhang J., Shi L. (2024). Novel subsets of peripheral immune cells associated with promoting stroke recovery in mice.. CNS Neurosci. Ther..

[57] Lai X., Ding H., Yu R., Bai H., Liu Y., Cao J. (2022). CXCL14 protects against polymicrobial sepsis by enhancing antibacterial functions of macrophages.. Am. J. Respir. Cell Mol. Biol..

[58] Chen X. (2021). ILT4 inhibition prevents TAM- and dysfunctional T cell-mediated immunosuppression and enhances the efficacy of anti-PD-L1 therapy in NSCLC with EGFR activation.. Theranostics.

[59] Pan J., Zhang L., Wang X., Li L., Yang C., Wang Z., Su K., Hu X., Zhang Y., Ren G., Jiang J., Li P., Huang J. (2023). Chronic stress induces pulmonary epithelial cells to produce acetylcholine that remodels lung pre-metastatic niche of breast cancer by enhancing NETosis.. J. Exp. Clin. Cancer Res..

[60] Lee S., Kim M.J., Ahn S.I., Choi S.K., Min K.Y., Choi W.S., You J.S. (2023). Epigenetic landscape analysis reveals the significance of early reduced chromatin accessibility in osteoclastogenesis.. Bone.

[61] Goc J., Germain C., Vo-Bourgais T.K.D., Lupo A., Klein C., Knockaert S., de Chaisemartin L., Ouakrim H., Becht E., Alifano M., Validire P., Remark R., Hammond S.A., Cremer I., Damotte D., Fridman W.H., Sautès-Fridman C., Dieu-Nosjean M.C. (2014). Dendritic cells in tumor-associated tertiary lymphoid structures signal a Th1 cytotoxic immune contexture and license the positive prognostic value of infiltrating CD8+ T cells.. Cancer Res..

[62] Tang H., Panse G., Braddock D., Perincheri S., Xu M.L., McNiff J.M. (2023). IRF8 may be a useful marker for blastic plasmacytoid dendritic cell neoplasm, especially with weak CD123 expression.. J. Cutan. Pathol..

[63] Martin E.M., Clark J.C., Montague S.J., Morán L.A., Di Y., Bull L.J., Whittle L., Raka F., Buka R.J., Zafar I., Kardeby C., Slater A., Watson S.P. (2024). Trivalent nanobody-based ligands mediate powerful activation of GPVI, CLEC-2, and PEAR1 in human platelets whereas FcγRIIA requires a tetravalent ligand.. J. Thromb. Haemost..

[64] Rotter Sopasakis V., Sandstedt J., Johansson M., Lundqvist A., Bergström G., Jeppsson A., Mattsson Hultén L. (2019). Toll-like receptor-mediated inflammation markers are strongly induced in heart tissue in patients with cardiac disease under both ischemic and non-ischemic conditions.. Int. J. Cardiol..

[65] Elenbaas J.S., Pudupakkam U., Ashworth K.J., Kang C.J., Patel V., Santana K., Jung I.H., Lee P.C., Burks K.H., Amrute J.M., Mecham R.P., Halabi C.M., Alisio A., Di Paola J., Stitziel N.O. (2023). Author Correction: SVEP1 is an endogenous ligand for the orphan receptor PEAR1.. Nat. Commun..

[66] Yang W.Y., Izzi B., Bress A.P., Thijs L., Citterio L., Wei F.F., Salvi E., Delli Carpini S., Manunta P., Cusi D., Hoylaerts M.F., Luttun A., Verhamme P., Hardikar S., Nawrot T.S., Staessen J.A., Zhang Z.Y. (2022). Association of colorectal cancer with genetic and epigenetic variation in PEAR1—A population-based cohort study.. PLoS One.

[67] Yao Y., Tang X.F., Zhang J. (2016). H Association of PEAR1 genetic variants with platelet reactivity in response to dual antiplatelet therapy with aspirin and clopidogrel in the Chinese patient population after percutaneous coronary intervention.. Thromb. Res..

[68] Zhong Y., Zhang Y., Wei S., Chen J., Zhong C., Cai W., Jin W., Peng H. (2022). Dissecting the effect of sphingolipid metabolism gene in progression and microenvironment of osteosarcoma to develop a prognostic signature.. Front. Endocrinol..

[69] Theresia K.J., Wolfgang H., Gundula G., Michael E., Alexander W., Caroline G., Laura F., Rabih C., Heinz-Peter G. (2023). Prenatal diagnosis of SMPD4 loss ‐ A neurodevelopmental disorder with microcephaly, arthrogryposis and structural brain anomalies.. Prenat. Diagn..

[70] Luan H., Chen S., Zhao L., Liu S., Luan T. (2023). Precise lipidomics decipher circulating ceramide and sphingomyelin cycle associated with the progression of rheumatoid arthritis.. J. Proteome Res..

[71] Li J., Manickam G., Ray S., Oh C., Yasuda H., Moffatt P., Murshed M. (2016). Smpd3 expression in both chondrocytes and osteoblasts is required for normal endochondral bone development.. Mol. Cell. Biol..

[72] Stoffel W., Hammels I., Jenke B., Schmidt-Soltau I., Niehoff A. (2019). Neutral sphingomyelinase 2 (SMPD3) deficiency in mice causes chondrodysplasia with unimpaired skeletal mineralization.. Am. J. Pathol..

[73] Liu F., Li X., Yue H., Ji J., You M., Ding L., Fan H., Hou Y. (2017). TLR ‐induced SMPD 3 defects enhance inflammatory response of B cell and macrophage in the pathogenesis of SLE.. Scand. J. Immunol..

[74] Liu-Bryan R. (2013). Synovium and the innate inflammatory network in osteoarthritis progression.. Curr. Rheumatol. Rep..

[75] Robinson W.H., Lepus C.M., Wang Q., Raghu H., Mao R., Lindstrom T.M., Sokolove J. (2016). Low-grade inflammation as a key mediator of the pathogenesis of osteoarthritis.. Nat. Rev. Rheumatol..

