# Supplementary material for: Electroacupuncture Ameliorates Knee Osteoarthritis By Rebalancing T Cell Homeostasis as Revealed By Immune Repertoire (IR) Sequencing
Source: Comb Chem High Throughput Screen. 2024 Aug 12;28(14):2546–63. doi: 10.2174/0113862073303471240805061026 (PMC12678985; doi:10.2174/0113862073303471240805061026)
Supplement: Supplementary file 1 [file CCHTS-28-14-2546_SD1.pdf]

Supplementary Material

Electroacupuncture Ameliorates Knee Osteoarthritis By Rebalancing T Cell Homeostasis as Revealed By Immune Repertoire (IR) Sequencing

Wenrui Jia[1,#], Yunan Zhang[2,#], Tianqi Wang[1], Cunzhi Liu[1], Jianfeng Tu[1], Guangxia Shi[1], LingYi Cai[1], Jingwen Yang[1,*] and Guangrui Huang[2,*]

[1]*School of Acupuncture-Moxibustion and Tuina, Beijing University of Chinese Medicine, Beijing 102488, China;* [2]*School of Life Science, Beijing University of Chinese Medicine, Beijing 102488, China*

Supplement Table 1. Baseline Demographic

Group	Age (years)		BMI		Sex-female (%)
	mean	SD	mean	SD	
Healthy control	62.00	4.58	22.96	0.81	100
KOA（Pre-treatment）	64.67	3.22	22.88	1.07	100
P-value	0.456		0.922		

Supplement Table 2 Change in WOMAC and NRS Scores.

		KOA（Pre-treatment）(week 0)	KOA（Post-treatment）(week 8)	
		Mean ± SD	Mean ± SD	*P*
WOMAC	Total	21.0 ± 10.02	10.3 ± 3.93	0.23
	Pain	5.3 ± 2.40	3.0 ± 1.53	0.19
	Stiffness	1.7 ± 0.33	1.0 ± 0.58	0.18
	Function	14.0 ± 8.00	6.3 ± 2.93	0.28
NRS		7.7 ± 0.33	3.7 ± 1.20	0.07

Supplement Table 3 Diversity of TCR β-chain CDR3 repertoire.

	Healthy control		KOA（Pre-treatment）		KOA（Post-treatment）		*P*	
	mean	SD	mean	SD	mean	SD	Con VS Pre-	Pre- VS Post-
CDR3	1137546.67	396175.616	902135.33	183130.325	757319	236758.561	0.403	0.362
Unique_CDR3	63231.67	25107.717	37849.33	17600.558	33441	15600.077	0.035	0.797
D50	9.8	3.2047	0.433	0.3786	7.033	5.2548	0.028	0.144
Diversity_Index	24.167	2.655811	14.333	3.9323	21	3.1177	0.046	0.009
Entropy	11.567	0.8737	8.267	1.4468	10.9	1.249	0.028	0.01

Supplement Table 4 72 Differential Genes in Pre-Treatment Patients Compared to a Healthy Population

ID	LogFC	Pvalue	Symbol	Pre-T O1-Pre	Pre-T O2-Pre	Pre-T O3-Pre	Healthy H1	Healthy H2	Healthy H3
ENSG00000171161	-3.30	1.1E-05	ZNF672	0.921	0.982	1.185	9.771	9.291	9.002
ENSG00000272170	-4.39	4.8E-04	AL355385.1	0.087	0.182	0.000	0.743	0.839	0.362
ENSG00000105507	5.04	7.1E-04	CABP5	1.360	0.336	0.167	0.043	0.000	0.000
ENSG00000280046	4.95	8.8E-04	AC104581.4	0.880	1.058	0.567	0.002	0.073	0.058
ENSG00000212464	-3.57	1.7E-03	SNORA12	0.242	0.381	0.000	0.762	0.778	0.896
ENSG00000183134	-3.18	3.0E-03	PTGDR2	0.147	0.206	0.786	3.299	1.558	1.769
ENSG00000284874	2.92	3.1E-03	AC000093.1	4.449	1.582	0.910	0.099	0.185	0.652
ENSG00000166091	3.45	3.5E-03	CMTM5	2.211	0.539	0.803	0.106	0.023	0.126
ENSG00000140263	-1.41	3.0E-03	SORD	1.389	1.106	0.907	3.563	2.746	2.668
ENSG00000274341	-3.64	5.1E-03	AC005899.6	0.179	0.126	0.169	1.023	0.550	1.497
ENSG00000152332	2.22	4.5E-03	UHMK1	3.091	3.505	1.667	0.548	0.196	1.574
ENSG00000128218	4.29	7.0E-03	VPREB3	0.931	0.245	1.536	0.000	0.000	0.108
ENSG00000174173	-1.85	6.5E-03	TRMT10C	0.564	0.474	0.594	1.661	1.255	1.814
ENSG00000103355	-2.55	5.8E-03	PRSS33	1.059	0.459	2.408	9.377	4.310	3.485
ENSG00000105366	-2.53	7.8E-03	SIGLEC8	0.392	0.118	0.464	2.119	1.314	0.748
ENSG00000269713	2.11	7.4E-03	NBPF9	0.714	0.357	1.071	0.078	0.148	0.173
ENSG00000284981	-2.51	1.0E-02	UPK3BL2	0.618	1.392	0.466	1.703	8.813	2.867
ENSG00000107263	1.32	7.3E-03	RAPGEF1	31.456	32.396	30.465	9.513	8.665	24.517
ENSG00000272144	-3.40	2.0E-02	AC025171.4	0.203	0.445	0.000	0.397	0.640	0.786
ENSG00000161905	-1.76	1.3E-02	ALOX15	1.147	0.866	1.735	7.571	2.583	2.574
ENSG00000260545	3.13	2.2E-02	AC026771.1	1.198	0.450	0.121	0.000	0.118	0.000
ENSG00000091181	-1.96	1.6E-02	IL5RA	0.167	0.220	0.477	1.239	0.926	0.489
ENSG00000081041	4.82	2.1E-02	CXCL2	47.296	3.035	1.071	0.288	0.207	0.077
ENSG00000103056	-1.71	1.6E-02	SMPD3	0.332	0.276	0.761	1.092	1.676	0.836
ENSG00000211899	1.91	1.4E-02	IGHM	52.653	12.783	18.208	5.823	3.035	14.845
ENSG00000158050	2.25	1.3E-02	DUSP2	250.980	48.068	29.711	14.622	23.310	11.549
ENSG00000187699	1.81	2.2E-02	C2orf88	1.385	0.563	0.190	0.152	0.120	0.121
ENSG00000126698	-0.98	1.7E-02	DNAJC8	6.652	5.245	4.901	11.599	13.857	9.482
ENSG00000215472	8.16	2.5E-02	RPL17-C18orf32	28.796	0.000	19.707	0.000	0.000	0.000
ENSG00000115008	4.23	3.1E-02	IL1A	3.527	0.061	0.000	0.000	0.000	0.000
ENSG00000207445	-3.01	3.4E-02	SNORD15B	1.581	0.000	0.000	0.767	1.119	1.015
ENSG00000105205	-1.76	1.8E-02	CLC	24.855	12.986	79.489	158.430	76.377	86.562
ENSG00000161911	2.05	2.5E-02	TREML1	7.566	2.868	0.733	0.582	0.353	0.777
ENSG00000259671	2.91	3.1E-02	MTCYBP23	5.993	7.009	0.395	0.174	0.341	0.433
ENSG00000090975	1.79	2.7E-02	PITPNM2	1.143	0.316	0.157	0.108	0.087	0.097

ENSG00000143786	1.81	2.7E-02	CNIH3	0.712	1.464	0.372	0.070	0.299	0.294
ENSG00000228166	2.75	4.1E-02	MTND1P11	0.466	1.235	0.229	0.037	0.168	0.038
ENSG00000187800	2.56	3.4E-02	PEAR1	1.124	0.553	0.061	0.052	0.041	0.048
ENSG00000263155	4.16	4.0E-02	MYZAP	2.322	0.029	0.052	0.000	0.000	0.026
ENSG00000267607	2.26	2.6E-02	AC011511.5	4.539	2.144	1.829	0.094	1.965	0.762
ENSG00000274286	4.51	4.2E-02	ADRA2B	2.223	0.089	0.050	0.000	0.092	0.000
ENSG00000127920	2.21	3.2E-02	GNG11	3.214	1.197	0.635	1.136	0.064	0.194
ENSG00000280347	2.33	3.7E-02	AC000123.3	0.878	1.110	0.094	0.039	0.079	0.146
ENSG00000224967	2.74	4.9E-02	AC009303.1	0.555	0.954	0.048	0.000	0.000	0.187
ENSG00000259379	2.91	3.5E-02	MTND5P32	5.606	7.433	0.297	0.266	0.245	0.329
ENSG00000124491	1.76	2.7E-02	F13A1	7.325	3.365	1.249	2.053	0.714	0.534
ENSG00000268758	-1.53	3.3E-02	ADGRE4P	0.871	0.994	0.111	1.228	1.353	1.031
ENSG00000177272	2.38	3.8E-02	KCNA3	3.386	1.525	0.202	0.100	0.134	0.359
ENSG00000124762	1.98	3.0E-02	CDKN1A	15.152	2.318	1.739	0.928	1.527	0.648
ENSG00000112759	-1.52	3.1E-02	SLC29A1	0.854	0.406	0.668	1.822	2.869	0.740
ENSG00000111886	2.99	4.5E-02	GABRR2	4.522	6.266	0.218	0.086	0.293	0.391
ENSG00000154146	1.78	3.1E-02	NRGN	82.707	19.454	10.979	14.054	5.068	6.994
ENSG00000140968	1.57	4.5E-02	IRF8	0.891	0.296	0.360	0.267	0.054	0.112
ENSG00000277969	1.83	3.5E-02	AC006449.6	15.859	3.088	1.931	1.728	0.641	1.743
ENSG00000109272	1.49	5.0E-02	PF4V1	6.088	2.287	1.570	1.226	0.689	0.505
ENSG00000119616	-1.33	3.6E-02	FCF1	0.759	1.749	0.532	1.206	2.836	2.931
ENSG00000275070	1.28	4.6E-02	RF00017	8.604	8.787	4.854	1.802	3.960	1.798
ENSG00000120885	1.90	3.8E-02	CLU	4.347	1.074	0.551	0.907	0.247	0.194
ENSG00000182557	-1.54	4.3E-02	SPNS3	0.873	0.285	0.923	2.122	1.778	0.853
ENSG00000090924	2.59	3.6E-02	PLEKHG2	9.118	5.307	0.462	0.134	0.821	0.947
ENSG00000183336	-1.37	5.0E-02	BOLA2	0.792	0.594	0.240	0.739	0.633	2.124
ENSG00000155090	2.16	3.4E-02	KLF10	48.276	8.449	4.309	1.826	7.499	1.621
ENSG00000122861	2.44	3.9E-02	PLAU	33.702	2.783	1.503	0.862	0.787	1.278
ENSG00000163736	2.04	4.0E-02	PPBP	54.453	16.673	7.628	17.921	2.080	2.372
ENSG00000100351	1.52	4.2E-02	GRAP2	6.000	1.730	0.873	1.101	0.491	0.694
ENSG00000105369	1.78	4.8E-02	CD79A	7.324	1.974	5.076	0.383	0.892	3.681
ENSG00000280194	1.78	3.8E-02	AD000864.1	61.918	18.265	6.000	4.105	9.598	5.195
ENSG00000101162	2.31	4.4E-02	TUBB1	29.586	6.745	1.187	2.393	0.799	1.097
ENSG00000131188	1.25	3.9E-02	PRR7	55.295	14.287	21.338	11.537	15.898	8.122
ENSG00000005961	1.96	5.0E-02	ITGA2B	7.164	1.882	0.606	1.412	0.244	0.368
ENSG00000118363	-1.00	4.9E-02	SPCS2	2.316	3.960	3.355	8.068	6.894	4.351
ENSG00000285106	0.95	4.6E-02	AC016831.7	4.499	3.786	2.505	1.636	3.105	1.271

Supplement Table 5. 72 Differential Genes in Post-Treatment Patients Compared to Pre-Treatment Population

ID	LogFC	Pvalue	Symbol	Post-T O1-Post	Post-T O2-Post	Post-T O3-Post	Pre-T O1-Pre	Pre-T O2-Pre	Pre-T O3-Pre
ENSG00000107263	-1.16	0.005	RAPGEF1	9.768	20.022	15.344	31.456	32.396	30.465
ENSG00000081041	-3.39	0.001	CXCL2	2.800	0.244	0.752	47.296	3.035	1.071
ENSG00000171161	2.45	0.003	ZNF672	8.867	9.778	1.435	0.921	0.982	1.185
ENSG00000284874	-2.16	0.002	AC000093.1	2.205	0.137	0.146	4.449	1.582	0.910
ENSG00000280194	-1.30	0.008	AD000864.1	19.813	5.145	5.757	61.918	18.265	6.000
ENSG00000154146	-1.45	0.008	NRGN	17.608	11.220	6.502	82.707	19.454	10.979
ENSG00000268758	1.90	0.003	ADGRE4P	3.779	2.175	0.731	0.871	0.994	0.111
ENSG00000122861	-1.89	0.009	PLAU	4.111	1.001	1.501	33.702	2.783	1.503
ENSG00000161905	1.91	0.008	ALOX15	6.518	6.162	2.114	1.147	0.866	1.735
ENSG00000103056	2.14	0.010	SMPD3	3.284	1.888	0.801	0.332	0.276	0.761
ENSG00000143786	-2.36	0.004	CNIH3	0.088	0.109	0.249	0.712	1.464	0.372
ENSG00000105205	1.97	0.018	CLC	298.674	98.321	56.980	24.855	12.986	79.489
ENSG00000285106	-1.05	0.018	AC016831.7	3.242	0.996	1.446	4.499	3.786	2.505
ENSG00000118363	1.16	0.016	SPCS2	8.223	6.124	7.789	2.316	3.960	3.355
ENSG00000101162	-1.95	0.017	TUBB1	2.438	2.449	1.750	29.586	6.745	1.187
ENSG00000120885	-1.64	0.013	CLU	0.760	0.576	0.318	4.347	1.074	0.551
ENSG00000211899	-1.38	0.020	IGHM	33.687	0.864	13.056	52.653	12.783	18.208
ENSG00000152332	-1.24	0.018	UHMK1	0.986	2.731	0.608	3.091	3.505	1.667
ENSG00000005961	-1.60	0.016	ITGA2B	1.140	1.017	0.453	7.164	1.882	0.606
ENSG00000090924	-1.44	0.020	PLEKHG2	2.473	0.917	0.890	9.118	5.307	0.462
ENSG00000155090	-1.35	0.023	KLF10	8.197	3.426	5.849	48.276	8.449	4.309
ENSG00000091181	1.93	0.013	IL5RA	1.519	1.033	0.668	0.167	0.220	0.477
ENSG00000124491	-1.47	0.021	F13A1	1.233	1.838	1.006	7.325	3.365	1.249
ENSG00000158050	-0.78	0.030	DUSP2	162.343	19.389	24.257	250.980	48.068	29.711
ENSG00000131188	-0.89	0.028	PRR7	27.854	9.437	11.767	55.295	14.287	21.338
ENSG00000163736	-1.29	0.023	PPBP	13.215	9.617	5.737	54.453	16.673	7.628
ENSG00000127920	-1.74	0.013	GNG11	0.800	0.612	0.134	3.214	1.197	0.635
ENSG00000161911	-1.62	0.016	TREML1	1.648	1.113	0.530	7.566	2.868	0.733
ENSG00000090975	-1.40	0.018	PITPNM2	0.484	0.078	0.104	1.143	0.316	0.157
ENSG00000187699	-2.82	0.007	C2orf88	0.039	0.135	0.068	1.385	0.563	0.190
ENSG00000267607	-1.15	0.027	AC011511.5	1.976	1.276	0.749	4.539	2.144	1.829
ENSG00000103355	2.13	0.024	PRSS33	10.921	4.619	1.538	1.059	0.459	2.408
ENSG00000124762	-1.26	0.032	CDKN1A	3.452	1.172	1.748	15.152	2.318	1.739
ENSG00000126698	0.85	0.037	DNAJC8	12.897	7.657	11.836	6.652	5.245	4.901
ENSG00000111886	-1.93	0.023	GABRR2	0.471	1.077	0.667	4.522	6.266	0.218

ENSG00000105369	-1.23	0.028	CD79A	5.515	0.244	2.479	7.324	1.974	5.076
ENSG00000280046	-1.21	0.030	AC104581.4	0.318	0.293	0.493	0.880	1.058	0.567
ENSG00000112759	1.41	0.032	SLC29A1	3.089	1.780	0.659	0.854	0.406	0.668
ENSG00000284981	2.52	0.017	UPK3BL2	9.620	0.274	6.853	0.618	1.392	0.466
ENSG00000140968	-1.93	0.014	IRF8	0.261	0.014	0.195	0.891	0.296	0.360
ENSG00000166091	-1.84	0.020	CMTM5	0.365	0.338	0.184	2.211	0.539	0.803
ENSG00000259671	-1.92	0.023	MTCYBP23	0.526	1.331	0.869	5.993	7.009	0.395
ENSG00000140263	0.92	0.045	SORD	2.572	2.659	1.673	1.389	1.106	0.907
ENSG00000269713	-2.04	0.031	NBPF9	0.087	0.405	0.068	0.714	0.357	1.071
ENSG00000280347	-2.12	0.019	AC000123.3	0.113	0.088	0.182	0.878	1.110	0.094
ENSG00000105366	2.13	0.025	SIGLEC8	2.550	1.486	0.453	0.392	0.118	0.464
ENSG00000100351	-0.89	0.048	GRAP2	3.334	0.607	0.790	6.000	1.730	0.873
ENSG00000119616	1.07	0.045	FCF1	0.921	3.287	2.700	0.759	1.749	0.532
ENSG00000182557	1.68	0.035	SPNS3	4.233	1.961	0.777	0.873	0.285	0.923
ENSG00000259379	-1.82	0.037	MTND5P32	0.487	1.516	0.996	5.606	7.433	0.297
ENSG00000263155	-5.40	0.001	MYZAP	0.000	0.000	0.000	2.322	0.029	0.052
ENSG00000215472	-8.40	0.001	RPL17-C18orf32	0.000	0.000	0.000	28.796	0.000	19.707
ENSG00000277969	-1.01	0.050	AC006449.6	5.760	2.300	1.228	15.859	3.088	1.931
ENSG00000177272	-1.43	0.034	KCNA3	1.159	0.278	0.346	3.386	1.525	0.202
ENSG00000187800	-1.90	0.026	PEAR1	0.102	0.172	0.071	1.124	0.553	0.061
ENSG00000272170	3.93	0.002	AL355385.1	0.881	0.403	0.418	0.087	0.182	0.000
ENSG00000183336	1.36	0.034	BOLA2	2.478	0.847	0.753	0.792	0.594	0.240
ENSG00000174173	1.35	0.038	TRMT10C	2.332	0.466	1.847	0.564	0.474	0.594
ENSG00000260545	-3.80	0.002	AC026771.1	0.113	0.000	0.000	1.198	0.450	0.121
ENSG00000115008	-3.86	0.007	IL1A	0.059	0.000	0.000	3.527	0.061	0.000
ENSG00000109272	-1.90	0.027	PF4V1	0.750	0.386	1.146	6.088	2.287	1.570
ENSG00000183134	2.01	0.047	PTGDR2	3.085	1.045	0.661	0.147	0.206	0.786
ENSG00000274286	-2.04	0.029	ADRA2B	0.335	0.044	0.061	2.223	0.089	0.050
ENSG00000228166	-3.42	0.007	MTND1P11	0.036	0.209	0.000	0.466	1.235	0.229
ENSG00000128218	-2.63	0.016	VPREB3	0.206	0.000	0.336	0.931	0.245	1.536
ENSG00000273975	2.62	0.022	RF00017	0.855	1.897	0.393	0.306	0.911	0.000
ENSG00000105507	-2.17	0.034	CABP5	0.167	0.049	0.091	1.360	0.336	0.167
ENSG00000272144	2.50	0.038	AC025171.4	0.838	0.490	0.326	0.203	0.445	0.000
ENSG00000212464	2.58	0.036	SNORA12	0.426	0.624	0.698	0.242	0.381	0.000
ENSG00000207445	2.83	0.042	SNORD15B	1.181	0.502	1.522	1.581	0.000	0.000
ENSG00000274341	2.40	0.042	AC005899.6	0.422	0.370	1.093	0.179	0.126	0.169
ENSG00000224967	-2.52	0.044	AC009303.1	0.000	0.052	0.049	0.555	0.954	0.048

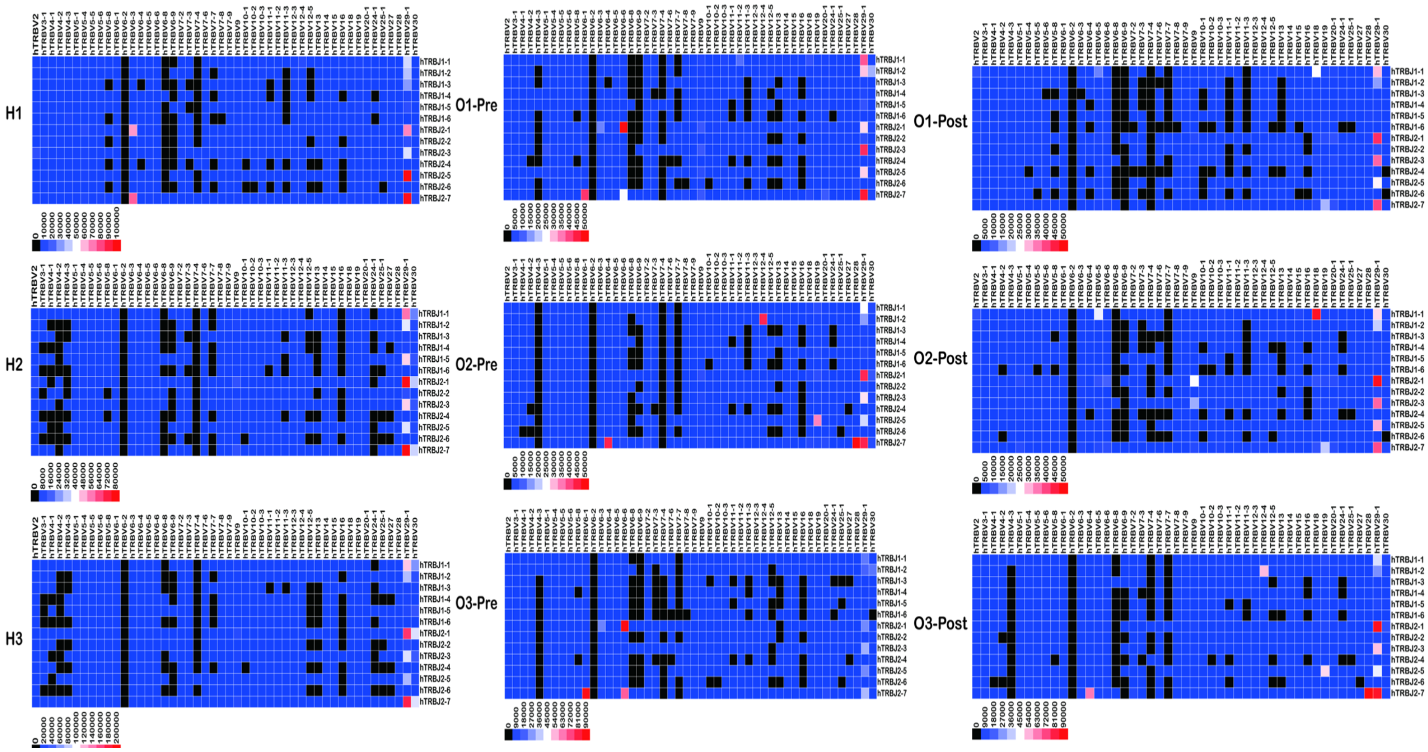

Supplement Figure 1 2D map of the frequency of V-J gene combinations from all samples. The relative frequency from low (black) to high (red) of V-J gene combinations is illustrated in the 2D heat maps from varying amounts of punch disks. Left to right: healthy control subjects, KOA subjects prior to treatment and KOA subjects posttreatment.

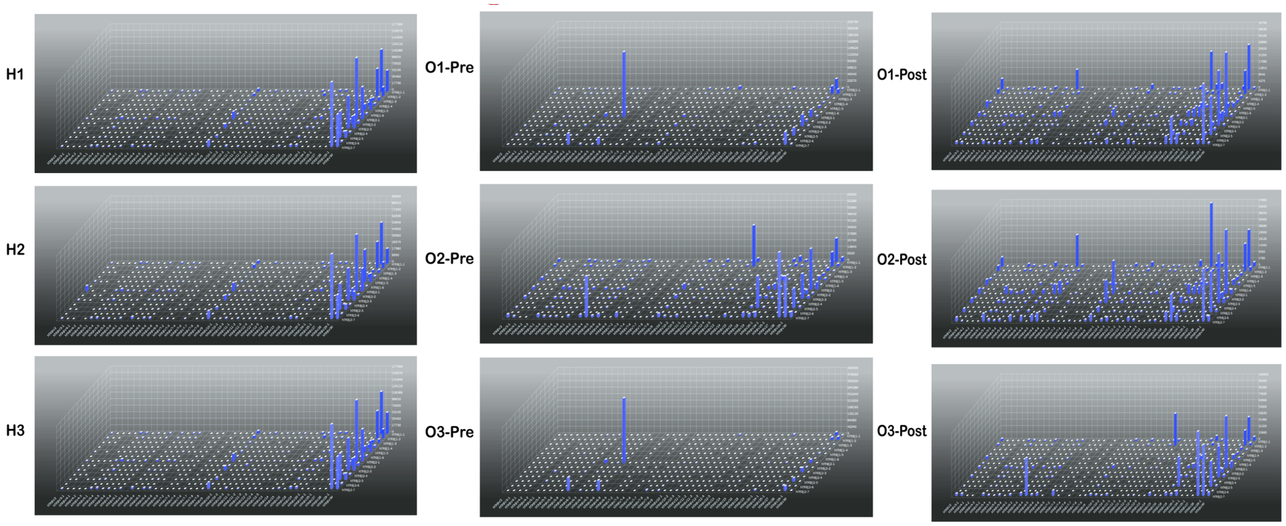

Supplement Figure 2 3D map of the frequency of V-J gene combinations from all samples. H1, H2, H3: Healthy control subjects; O1-Pre, O2-Pre, O3-Pre: KOA subjects prior to treatment; O1-Post, O2-Post, O3-Post: KOA subjects post to treatment.

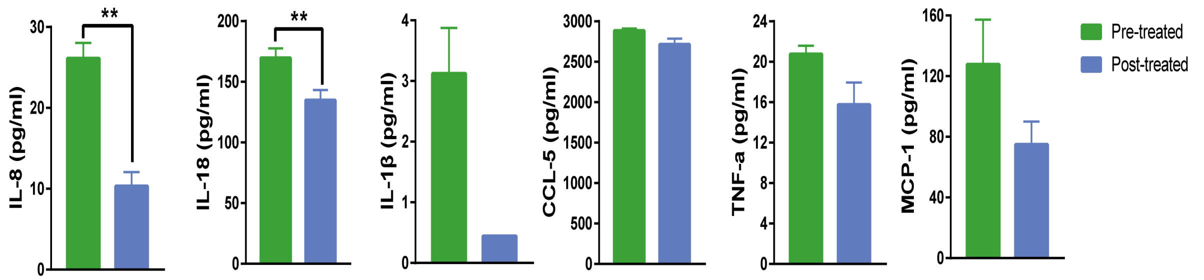

Supplement Figure 3 Comparison of plasma pro-inflammatory cytokine concentrations. *Intra-group comparison: pre versus post treatment, ***$p < 0.01$.**

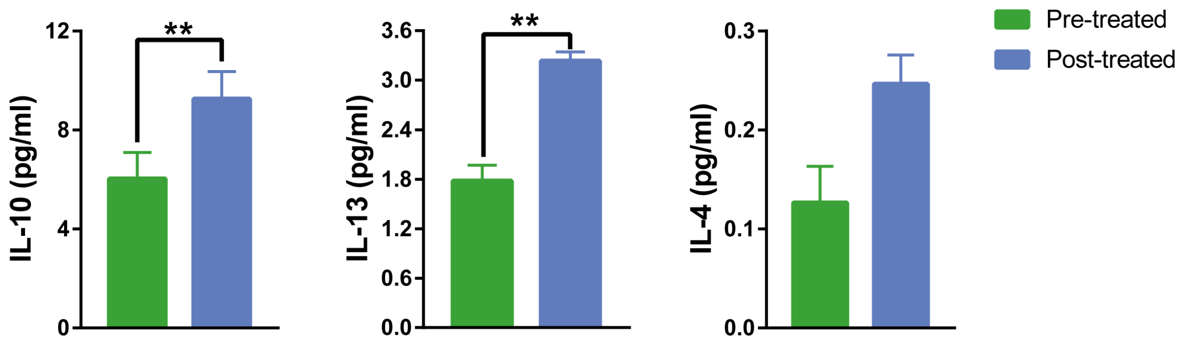

Supplement Figure 4 Comparison of plasma anti-inflammatory cytokine concentrations. *Intra-group comparison: pre versus post treatment, ***$p < 0.01$.**

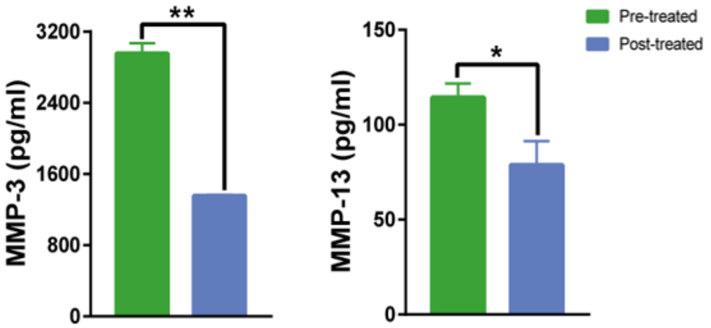

Supplement Figure 5 Comparison of plasma cartilage degradation biomarkers. *Intra-group comparison: pre versus post treatment, *$p < 0.05$, **$p < 0.01$.

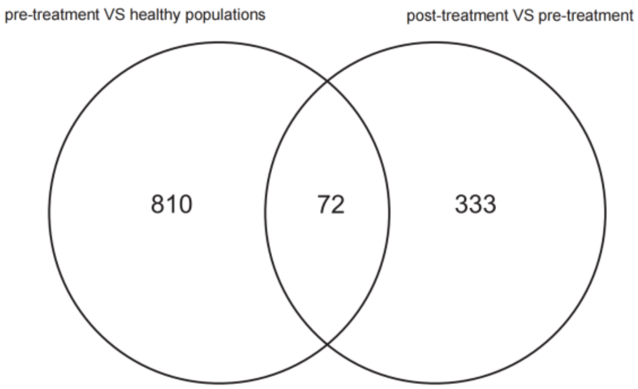

Supplement Figure 6 Vene diagrams between the three group.
